# Macrophages, Nitric Oxide and microRNAs Are Associated with DNA Damage Response Pathway and Senescence in Inflammatory Bowel Disease

**DOI:** 10.1371/journal.pone.0044156

**Published:** 2012-09-06

**Authors:** Jane J. Sohn, Aaron J. Schetter, Harris G. Yfantis, Lisa A. Ridnour, Izumi Horikawa, Mohammed A. Khan, Ana I. Robles, S. Perwez Hussain, Akiteru Goto, Elise D. Bowman, Lorne J. Hofseth, Jirina Bartkova, Jiri Bartek, Gerald N. Wogan, David A. Wink, Curtis C. Harris

**Affiliations:** 1 Laboratory of Human Carcinogenesis, Center for Cancer Research, National Cancer Institute, Bethesda, Maryland, United States of America; 2 Pathology and Laboratory Medicine, Baltimore Veterans Affairs Medical Center, and Department of Pathology, University of Maryland School of Medicine, Baltimore, Maryland, United States of America; 3 Radiation Biology Branch, National Cancer Institute, Bethesda, Maryland, United States of America; 4 Department of Pharmaceutical and Biomedical Sciences, South Carolina College of Pharmacy, University of South Carolina, Columbia, South Carolina, United States of America; 5 Cancer Society Research Center, Copenhagen, Denmark; 6 Institute of Molecular and Translational Medicine, Faculty of Medicine and Dentistry, Palacky University, Olomouc, Czech Republic; 7 Department of Biological Engineering, Center for Environmental Health Sciences, Massachusetts Institute of Technology, Cambridge, Massachusetts, United States of America; Institut Pasteur de Lille, France

## Abstract

**Background:**

Cellular senescence can be a functional barrier to carcinogenesis. We hypothesized that inflammation modulates carcinogenesis through senescence and DNA damage response (DDR). We examined the association between senescence and DDR with macrophage levels in inflammatory bowel disease (IBD). *In vitro* experiments tested the ability of macrophages to induce senescence in primary cells. Inflammation modulating microRNAs were identified in senescence colon tissue for further investigation.

**Methodology/Principal Findings:**

Quantitative immunohistochemistry identified protein expression by colon cell type. Increased cellular senescence (HP1γ; P = 0.01) or DDR (γH2A.X; P = 0.031, phospho-Chk2, P = 0.014) was associated with high macrophage infiltration in UC. Co-culture with macrophages (ANA-1) induced senescence in >80% of primary cells (fibroblasts MRC5, WI38), illustrating that macrophages induce senescence. Interestingly, macrophage-induced senescence was partly dependent on nitric oxide synthase, and clinically relevant NO• levels alone induced senescence. NO• induced DDR *in vitro*, as detected by immunofluorescence. In contrast to UC, we noted in Crohn’s disease (CD) that senescence (HP1γ; P<0.001) and DDR (γH2A.X; P<0.05, phospho-Chk2; P<0.001) were higher, and macrophages were not associated with senescence. We hypothesize that nitric oxide may modulate senescence in CD; epithelial cells of CD had higher levels of NOS2 expression than in UC (P = 0.001). Microarrays and quantitative-PCR identified miR-21 expression associated with macrophage infiltration and NOS2 expression.

**Conclusions:**

Senescence was observed in IBD with senescence-associated β-galactosidase and HP1γ. Macrophages were associated with senescence and DDR in UC, and *in vitro* experiments with primary human cells showed that macrophages induce senescence, partly through NO•, and that NO• can induce DDR associated with senescence. Future experiments will investigate the role of NO• and miR-21 in senescence. This is the first study to implicate macrophages and nitrosative stress in a direct effect on senescence and DDR, which is relevant to many diseases of inflammation, cancer, and aging.

## Introduction

Inflammatory bowel disease (IBD) is associated with high morbidity, poor quality of life and an increased risk of colon cancer in over 3.5 million people in the United States and Europe, with a steadily growing prevalence in Asia [1]. The most important risk factors for colon cancer development in IBD patients are duration and extent of inflammation. Patients with ulcerative colitis (UC), a subtype of IBD, develop colon cancer with a five-fold overall relative risk compared to population controls [2]. Colon tissue from IBD patients has been used to study the relationship between inflammation and cancer, with an emphasis on DNA damage. IBD is associated with increased etheno-DNA adducts [3], microsatellite instability [4], p53 mutational load [5] and clonal expansion of cells with mutations in polyguanine tracts [6]. UC tissues show initial activation of p53 in response to nitric oxide (NO•) [7], and eventual inactivation of p53 with increasing mutation load [5], resulting in a pattern of mutation unique compared to spontaneous colon cancer [8].

Evidence suggests that senescence acts as a barrier to carcinogenesis in UC and that this barrier is reduced in dysplastic lesions [9]. Inflamed colons from UC patients have increased expression of the DNA damage response pathway (DDR) sensor protein γH2A.X [10], which leads to activation of the stress-associated p53 pathway. DDR is implicated in the induction of premature cellular senescence [11], [12], independently of telomere length, which classically regulates senescence [13] in cellular aging. Prosenescent cytokines [14], WNT16 [15], and the Rb/p16 [16] pathway (through its induction of heterochromatin formation with HP1γ positive foci [17]), have all been implicated in premature cellular senescence. Premature cellular senescence halts carcinogenesis by limiting the proliferation of cells in the early stages of carcinogenesis [12], [18]–[20]. Senescence during inflammation is not well studied, but experiments *in vitro* have shown increased p53 and p21, in response to oxidative stress induced senescence [21], [22]. Elucidating the cause and outcome of inflammation-associated senescence is relevant for the 25% of human cancers associated with chronic inflammation and infection [23], [24].

Macrophages are a key component of a chronic inflammatory response and constitute part of the heterogeneous population of cells in tumors. Macrophages and NO• has been implicated in the activation of p53 [7] in IBD and the activation of the Akt pathway in breast cancer [25]. In addition, tumor-associated macrophages are implicated in carcinogenesis [26], [27]. We hypothesized that macrophages accelerate cellular senescence in epithelial cells at risk for carcinogenesis through the DNA damage pathway, in a NO• -dependent manner. NO• secreted by macrophages rapidly decreases in concentration with diffusion [28], thus cells may be exposed to different levels of NO• depending on distance from an NO• producing macrophage [29]. Stromal fibroblasts can be cellular targets of NO• and become senescent and secrete pro-inflammatory cytokines such as IL-6 and IL-8 [30]. We quantified macrophages in the lamina propria using quantitative immunohistochemistry (IHC) to identify macrophage numbers within the mucosa (i.e. macrophage infiltration). Levels of macrophage infiltration were correlated to DDR and senescence. Normal colonic epithelial cells can produce endogenous NO•, thus we also measured levels of NOS2 by IHC in the epithelium. Further, we determined if macrophages and NO• induce cellular senescence *in vitro*.

MicroRNAs (miRs) have been shown to be involved in nearly every biological process examined, including inflammation and senescence. To investigate the potential for miRs to be involved in macrophage or NOS2- induced senescence, we also evaluated the association of microRNAs with macrophage infiltration and NOS2 in IBD, and colonic adenomas.

## Methods and Patients

### Ethics Statement

This study was approved by the Institutional Review Board of the National Cancer Institute (OHSRP 3637, OHSRP 3961).

### Tissues

Colon tissues from UC and CD patients and colon adenomas were obtained from the Cooperative Human Tissue Network (Philadelphia, PA; Table S1). Two samples with varying degrees of gross inflammation were taken from each patient. Normal colons were obtained from University of Maryland, with tissues collected within 2 hours of death from patients who died of traumatic causes, were donors for organ transplants, and had no diseases related to the colon or chronic inflammation (Department of Pathology, University of Maryland, Baltimore, MD). Consent for the use of the tissues for research purposes was provided by next of kin or legally responsible individual on behalf of the deceased prior to the autopsy being performed. Investigators were not provided with any personal identifiers for these tissues and all patients were anonymous. Detailed clinical history was not provided, and the information on the extent of disease involvement in the small and large bowels was limited.

Tissues for IHC were fixed in 10% neutral buffered formalin, and embedded in paraffin. Samples without epithelial cells were excluded. A total of 29 UC colons, and 32 CD colons, and 5 normal colons met these criteria.

### Immunohistochemical Analysis

Immunohistochemistry (IHC) for DDR markers γH2A.X, phospho-Chk2, p53, and p21^WAF1^ was quantified by counting the number of positive epithelial cells versus total epithelial cells in three 250× magnification fields. An average of 3214 (UC) and 3178 epithelial cells (CD) were counted per sample in a blinded fashion by H. Y, a board certified pathologist. IHC for the monocyte and macrophage marker, CD68, was quantified by counting the number of stromal cells in the lamina propria. IHC for NOS2 was quantified by counting the number of epithelial and stromal cells in the lamina propria. Percent positivity was calculated by dividing the number of positive cells over total cells for each enumerated marker. For HP1γ, a combined score of intensity and distribution was used to score staining on a scale of 1–4 [31] to reflect the marked differences in both intensity and number of positive cells between UC and CD. All antibodies and further details are available in Materials and Methods S1. Antibodies for total Chk2 were tested by immunoblot for specificity (Figure S1) as described in the Materials and Methods S1.

### Coculture and Cell Culture Treatment

Normal human fibroblast strains MRC-5 and WI-38 (Coriell Institute for Medical Research, Camden, NJ), and murine macrophage strain ANA-1 [32] were grown in phenol red-free DMEM supplemented with 10% FBS (Biofluids, Rockville, MD), 4 mM glutamine (Biofluids), penicillin (10 units/ml), and streptomycin (10 µg/ml, Biofluids).

Cocultures were established by seeding 2500 normal human fibroblasts and 833 macrophages per well (3∶1 ratio) in a 6-well dish with 2 mL of media and cultured for 7 days. 200 µL of media was removed and replaced each day to replenish media contents. Fibroblasts were exposed to spermine NONOate (Sper/NO•; Sigma-Aldrich, St. Louis) as a NO• donor, or hydrogen peroxide (control) overnight (16 hrs) to evaluate induction of senescence in normal human fibroblasts. All experiments were repeated three times with three technical replicates for each repetition. At least 1500 cells were evaluated for senescence in each repetition using senescence-associated β-galactosidase (SA β-gal) buffer at pH 6.0 [33]. See Materials and Methods S1 for further details.

## Results

### UC and CD have Increased Macrophage Infiltration Compared to Normal Tissues

Both UC and CD had increased densities of macrophages, indicated by CD68+ cells, when compared to normal colons (*P*<0.05; Figure S2), reflective of the increased inflammation expected in UC and CD. UC and CD colons showed similar numbers of macrophages (*P*>0.05) compared to each other. The number of CD68+ cells was used to stratify tissues for this study; colons with macrophage numbers above the median were defined as having “high macrophage index”.

### Macrophage Infiltration is Positively Associated with Cellular Senescence in UC

**Figure 1:**
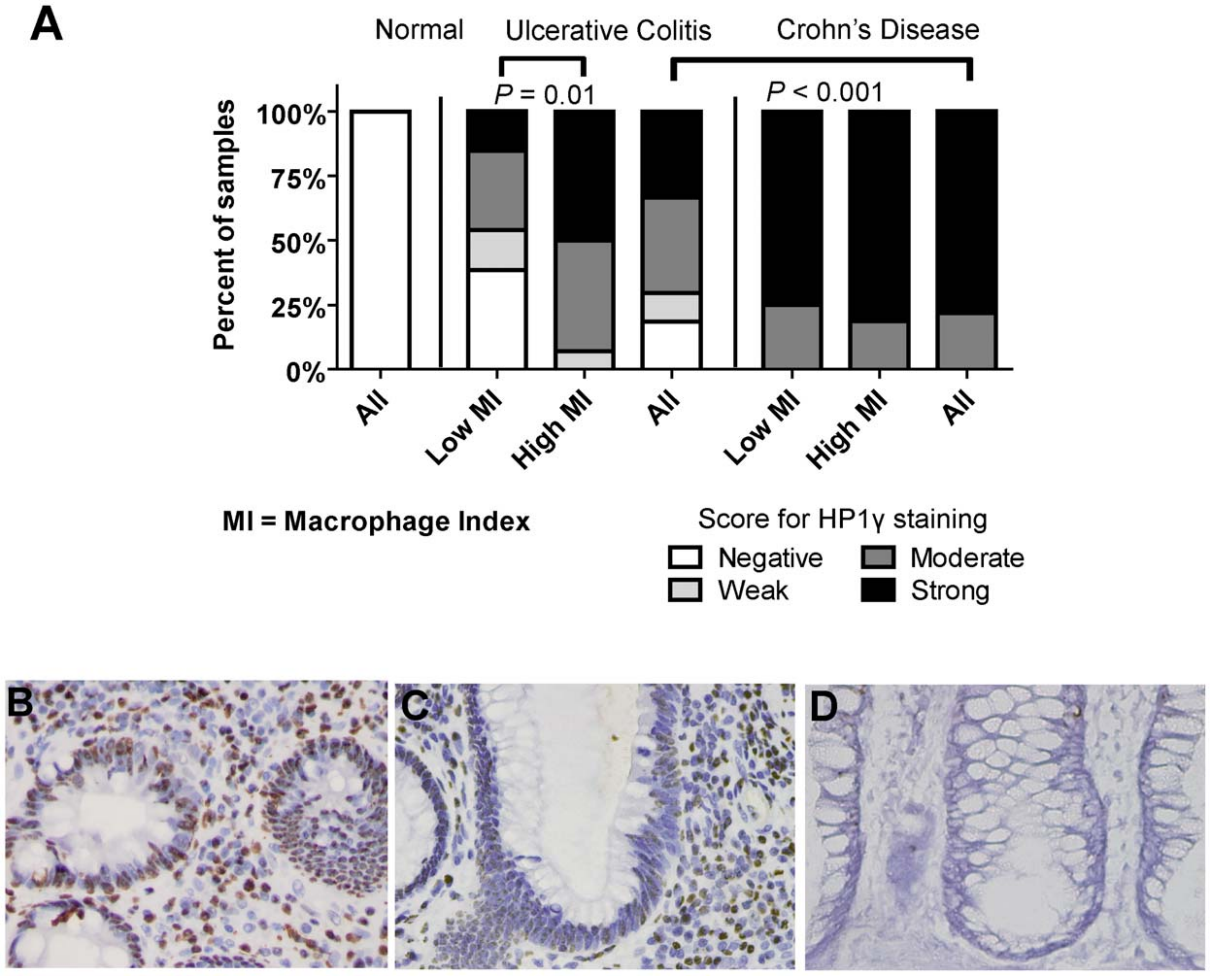
Senescence is induced in inflammatory bowel disease colons in association with infiltrating macrophages. Senescent epithelial cells were identified by HP1γ immunohistochemistry. Categorical scores reflecting intensity and distribution are shown as negative, weak, moderate, and strong. A) Colons from normal patients were negative for senescence-associated HP1γ. Ulcerative colitis and Crohn’s disease colonic epithelial cells were positive for HP1γ. Crohn’s disease colons had a greater percentage of HP1γ positive cells than ulcerative colitis colons (P<0.001). HP1γ was associated with high macrophage index (*P* = 0.01) in ulcerative colitis colons, but no such difference was observed within Crohn’s disease. B) A representative picture of a Crohn’s colitis crypt with strong HP1γ, C) a representative picture of ulcerative colitis crypt with weak staining, and D) a representative picture of normal autopsy tissue with negative staining. All are shown at 400× magnification. Epithelial and stromal HP1γ positive staining cells are shown in brown, with blue-purple hematoxylin counter stain in surrounding cells.

We measured HP1γ as an indicator of cellular senescence in formalin-fixed paraffin-embedded (FFPE) tissue. HP1γ localizes to senescence-associated heterochromatin foci *in vitro* [17], and correlates to SAβ-gal [33] in fresh colonic adenomas [12]. High macrophage index was associated with elevated staining for HP1γ in colonic epithelial cells of UC patients (*P* = 0.01). Macrophages in UC correlated with HP1γ in epithelial cells (*P* = 0.025; *Spearman*  = 0.43), indicating that macrophage infiltration is associated with senescence in nearby epithelial cells. In contrast, CD colonic epithelial cells had higher levels of HP1γ than UC (*P*<0.001; Figure 1A). HP1γ was not associated with high macrophage index in CD patients (Figure 1A), suggesting there may be other factors contributing to senescence in CD versus UC. Examples of strong HP1γ in CD, moderate staining in UC, and negative staining in normal tissues is shown (Figure 1B–D). Strong staining in colon adenoma (positive control) is shown in Figure S3E.

We next examined senescence-associated β-galactosidase **(**SAβ-gal) activity in frozen sections of UC and CD patients to confirm the presence of cellular senescence because enzyme activity is considered the gold standard. Fresh tissue is optimal for testing enzyme activity, but only archival frozen tissue was available for this study. Long-term storage of archival tissue may degrade enzyme activity, leading to false negatives, yet we were able to detect SAβ-gal activity in 13/21 (62%) UC and in 14/38 (37%) CD colons, illustrating for the first time that SAβ-gal-associated senescence is present in IBD tissue (Figure S3A-D). Immortalized normal human fibroblasts treated with Nutlin-3A [34] were used as positive controls.

### Activation of DDR (γ-H2A.X and Phospho-Chk2) is Higher in CD and UC than in Normal Tissue

**Figure 2:**
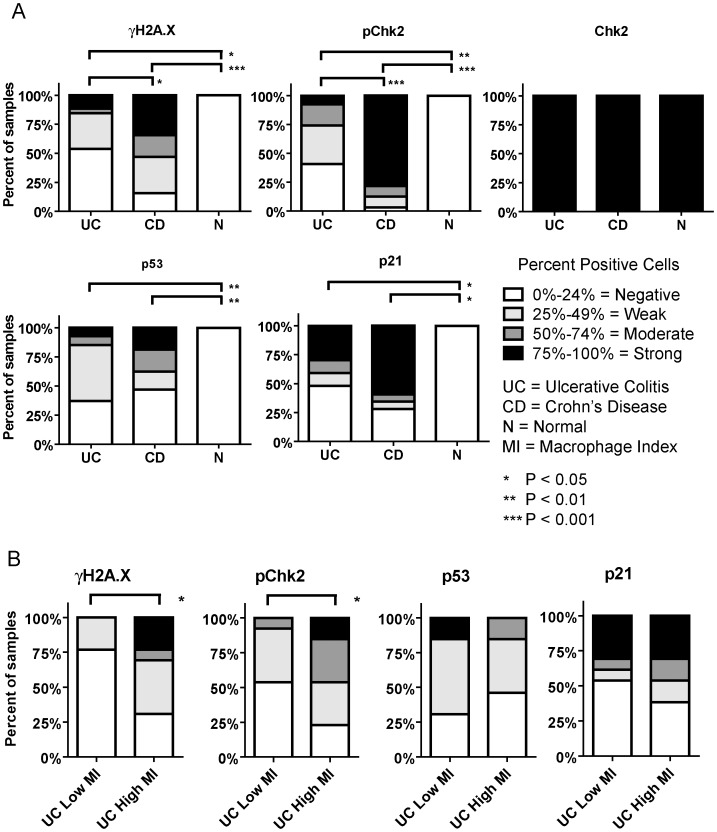
DNA damage response pathway and p21 are upregulated in inflammatory bowel disease and the DNA Damage response pathway is associated with high macrophage infiltration in ulcerative colitis. (A) Normal, ulcerative colitis, and Crohn’s disease colons were analyzed by immunostaining to determine the percent of positive epithelial cells for γ-H2A.X, phospho-Chk2, Chk2, p53 and p21. Data is shown by the percent of total samples with 0–24%, 25–49%, 50–74%, and 75–100% cell positivity. Normal colonic epithelial cells had low, or 0–24% cell positivity, for all markers. Both Crohn’s disease and ulcerative colitis colons had increased levels of γ-H2A.X (*P*<0.001; *P*<0.05), phospho-Chk2 (*P*<0.01; *P*<0.001), p53 (*P*<0.01) and p21 (*P*<0.05) compared to normal colon. Tissues from Crohn’s disease patients showed higher levels of γH2A.X (*P*<0.05), phospho-Chk2 (*P*<0.001) than in ulcerative colitis. No differences were detected in levels of total Chk2 between ulcerative colitis, Crohn’s disease, and normal colons, as expected. (B) Analysis of γH2A.X, phospho-Chk2, p53, and p21 in ulcerative colitis colonic epithelial cells was stratified by macrophage infiltration index to determine if macrophage infiltration in the lamina propria was associated with induction of the DNA damage response pathway and p21 activation. Colons with macrophage numbers above the median were defined as having high macrophage index, while those with macrophage numbers below the median were defined as having low macrophage index (i.e., low cellular densities). High macrophage index was associated with increased γH2A.X (*P* = 0.031) and phospho-Chk2 (*P* = 0.014). No significant differences were observed for p53 and p21 with respect to macrophage index.

We determined levels of DDR markers associated with premature senescence [11], [12] by IHC in the epithelial cells of IBD and normal colons. UC and CD colons showed increased levels of γH2A.X (*P*<0.05; *P*<0.001), phospho-Chk2 (*P*<0.01, *P*<0.001), p53 (*P*<0.01), and p21 (*P*<0.05) when compared to normal colons (Figure 2A). No increase was observed in total Chk2 in IBD versus normal colons, consistent with previous data that Chk2 is unchanged during colon carcinogenesis [11].

We found that UC colons had lower levels of DDR compared to CD, based on γH2A.X (*P*<0.05) and phospho-Chk2 (*P*<0.001) staining. No differences were observed in total Chk2, p53 or p21 between CD and UC (Figure 2A). Examples of staining patterns are shown in Figure S4.

### Macrophages are Positively Associated with Activation of DDR in UC

We examined if high macrophage index was associated with activation of DDR. In UC patients, high macrophage index was associated with increased γH2A.X (*P* = 0.031) and phospho-Chk2 (*P* = 0.014; Figure 2B) in colonic epithelium. No significant differences were observed for p53, or p21, although p21 was marginally increased in tissues with higher macrophage index (Figure 2B). In colons from CD patients, macrophage index was not associated with either activation of the DDR pathway or immunopositivity of p21 (Figure S5).

We hypothesized that macrophages directly induce senescence, based on the data from UC tissues. To test this, we performed the following *in vitro* experiments with macrophages and primary human cells.

### Macrophages cause NO• Induced Cellular Senescence *in vitro*

**Figure 3:**
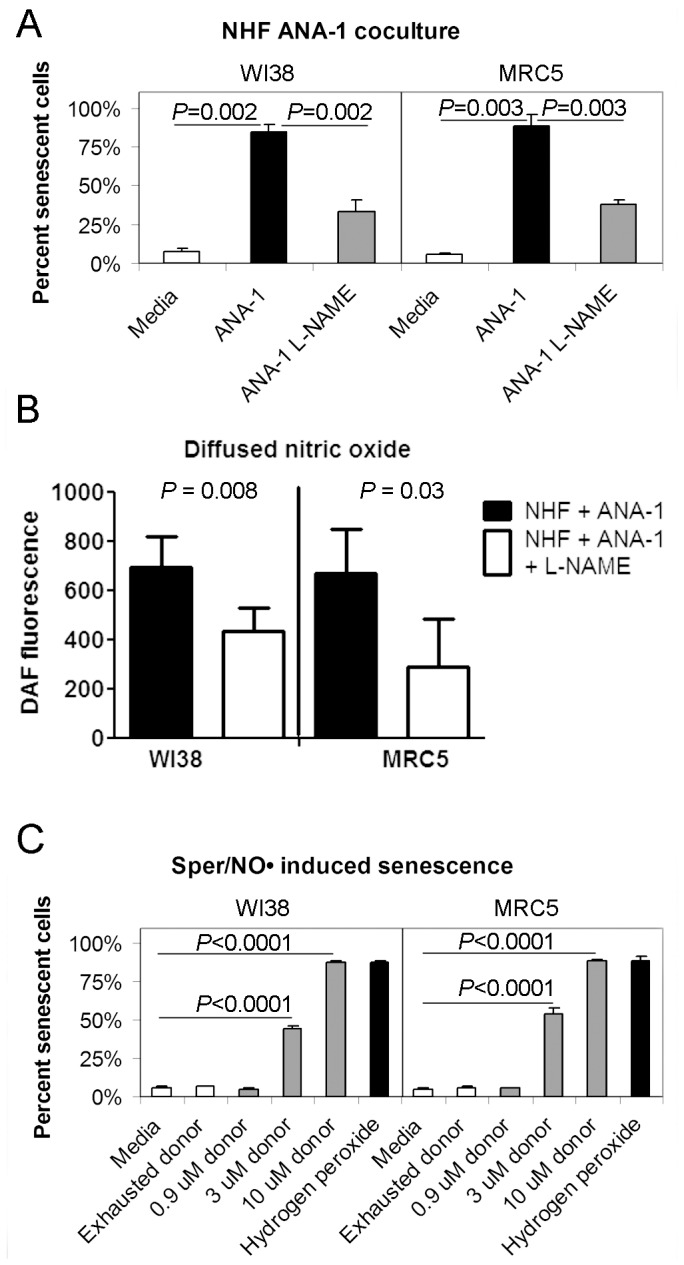
Senescence is induced by either macrophages or NO• in primary normal human fibroblasts in culture. Normal human fibroblasts (WI38 and MRC5) were grown in coculture with murine macrophages (ANA-1), or with the NO• donor spermine NONOate (Sper/NO•). Senescence-associated β-galactosidase activity was used to determine to the percent of senescent fibroblasts divided by the number of total fibroblasts. Results are shown from three experiments, with each experiment done in triplicate. (A) Normal human fibroblasts were cocultured with macrophages, with and without the NO• synthase inhibitor L-NAME (500 µM). Macrophages induced senescence in WI38 and MRC5 cells. Senescence was partially abrogated by L-NAME in WI38 and MRC5 cells. (B) The NO• synthase inhibitor L-NAME reduces diffused NO• in media of cocultures comprised of normal human fibroblasts (WI38 or MRC5) and macrophages (ANA-1). 100 µl of media from three separate cocultures was aliquoted with 100 µL of 5 µM of DAF in 96-well plates. Plates were read for DAF-fluorescence as an indicator of NO•. Addition of the NO• inhibitor L-NAME resulted in decreased levels of NO• in both WI38 (*P* = 0.008) and MRC5 (*P* = 0.03) cells. Fluorescence measurements from cocultures were normalized by subtracting the DAF fluorescence measured in media from wells with fibroblasts only. (C) Fibroblasts were dosed with 0.09 µM, 3 µM and 10 µM Sper/NO•, Sper/NO• that was previously incubated in media with sodium hydroxide (vehicle) for 48 hours (exhausted donor), and media alone (negative control) overnight (16 hrs). These concentrations were selected to achieve steady state concentrations of 4.5 nM, 15 nM and 50 nM NO• respectively. 10 µM and 3 µM Sper/NO• induced significant levels of senescence (*P*<0.0001). Exhausted Sper/NO• (negative control) and 0.09 µM Sper/NO• did not induce significant levels of senescence when compared to media alone. Hydrogen peroxide (200 µM; 2 hrs) was used as a positive control.

To investigate the role of macrophages in the induction of senescence *in vitro*, normal, primary human fibroblast strains MRC5 and WI38 were cocultured with macrophages for 7 days and evaluated by the SA-βgal assay. Fibroblasts are relevant because senescent stromal cells can produce proinflammatory cytokines that may influence the senescent state of epithelial cells. Approximately eighty percent of fibroblasts cocultured with macrophages were positive for SA-βgal, and showed more senescent blue-stained cells compared to fibroblasts grown alone (Figure 3A; WI38, *P* = 0.002; MRC5, *P* = 0.003).

To determine if NO• produced by macrophages may be capable of inducing senescence in stromal fibroblasts, macrophages and fibroblasts were cocultured in media with and without the NO• synthase inhibitor *N*-nitro-l-arginine methyl ester (L-NAME, 500 µM, Sigma-Aldrich, St. Louis). DAF-FM diacetate (4-amino-5-methylamino-2′, 7′-difluorofluorescein diacetate; DAF, Invitrogen, Carlsbad) was used to assess the amount of NO• diffused into the media of cocultured cells. As expected, L-NAME led to decreased NO• present in the media of cocultures (Figure 3B; WI38, *P* = 0.008, MRC5; *P* = 0.03). After exposure to coculture, fibroblasts were fixed and stained for SA-βgal activity at pH 6.0, resulting in blue substrate in senescent cells (Figure S6.) Cocultures grown in the presence of L-NAME showed decreased blue SA-βgal positive cells (Figure 3A; WI38, *P* = 0.002; MRC5, *P* = 0.003). This suggested that NO• is at least partially responsible for macrophage-induced senescence.

To determine if NO• alone could induce cellular senescence, normal human fibroblasts were exposed to clinically relevant levels of NO• and examined for SA-βgal activity. To achieve target steady state levels of 4.5 nM, 15 nM and 50 nM NO•, fibroblasts were incubated with 0.9 µM, 3 µM and 10 µM of the NO• donor Spermine NONOate (Sper/NO•). These doses were chosen because they are consistent with known levels of steady state NO• secreted by macrophages in vitro [35], [36] and levels of NO• detected in ulcerative colitis [37]. NO• concentrations at or below 50 nM are below the limit of detection for our NO• gas analyzer. To confirm that Sper/NO• was producing NO• levels near our target concentration, we measured NO• produced by 100 µM Sper/NO• (expected concentration of 500 nM NO•) and found steady state levels of 380 nM NO• at 4 hours (Figure S7; SD ±35 nM; n = 3), similar to the expected concentrations calculated from our previously published data [29]. Treatment with 3 µM and 10 µM, but not 0.9 µM, Sper/NO• induced enlarged SA-βgal positive cells (*P*<0.0001; Figure 3C; Figure S6). Thus, levels of NO• that are physiologically relevant to IBD induce senescence in a dose-dependent manner.

To determine if DDR is upregulated in cells induced into senescence by NO•, we performed immunofluorescence for γH2A.X in MRC5 cells treated with 10 µM Sper/NO•. Indeed, Sper/NO• treated cells showed increased levels of γH2A.X foci, compared to untreated control cells (Figure S8.).

### Higher Levels of NOS2 Expression in CD Correlates with Higher Levels of Senescence-associated HP1γ

**Figure 4:**
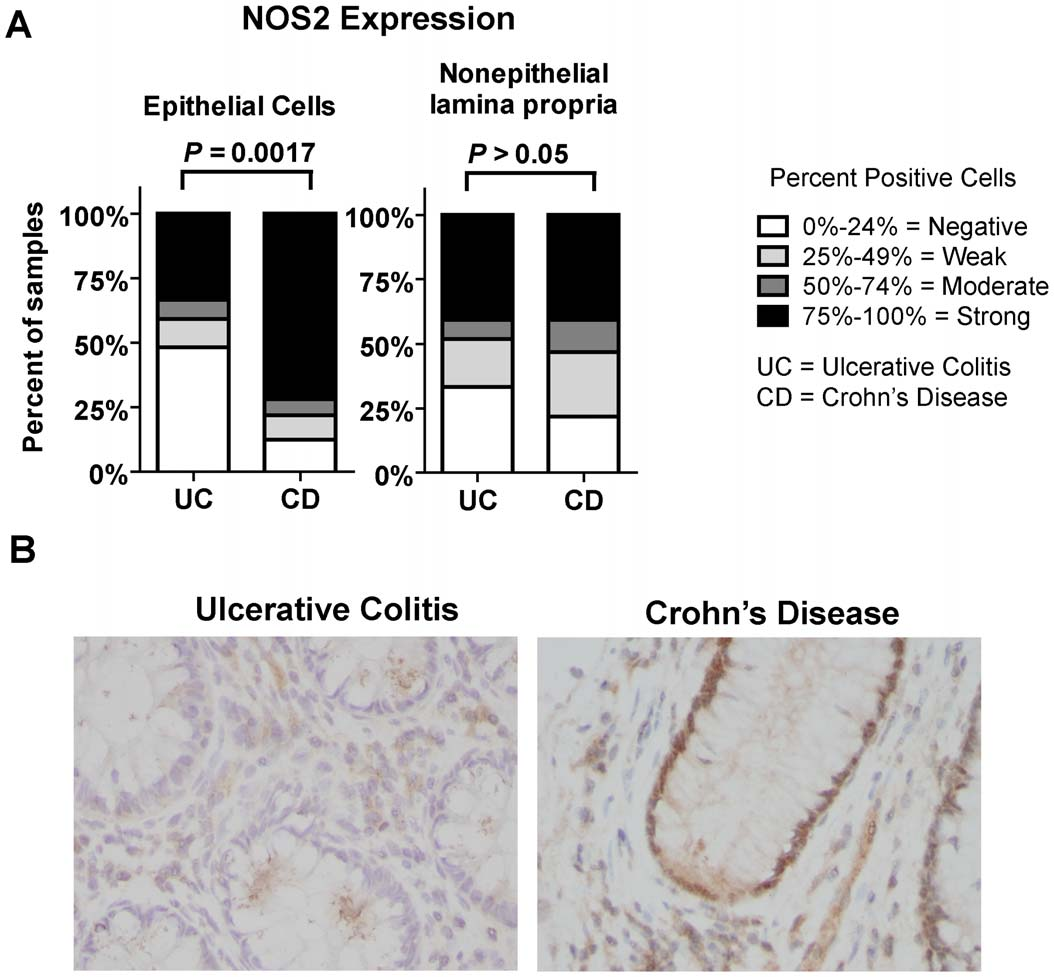
Epithelial cells in Crohn’s disease colon show higher levels of anti-NOS2 immunoreactivity than epithelial cells in ulcerative colitis colon. Immunohistochemistry for NOS2 was performed as a possible indicator of NO• produced in the colon of ulcerative colitis and Crohn’s disease patients. (A) Colonic epithelial cells had higher NOS2 expression in ulcerative colitis than Crohn’s disease (*P* = 0.0013) colons as shown by the percent of samples with positive cells while there was no significant difference in NOS2 expressing cells in the lamina propria. (B) Representative pictures show an ulcerative colitis section with low (0–24% positive) epithelial NOS2, and a Crohn’s disease section with high (75–100% positive) epithelial NOS2.

Macrophages were associated with senescence in epithelial cells in UC, but thus far we could not identify a driver of senescence in CD. Based on our *in vitro* studies that identified NO• as an inducer or senescence, we hypothesized that NO•, secreted by macrophages and produced by epithelial cells themselves may modulate senescence. To study this model of extracellular and intracellular-induced senescence, IHC for NOS2 was performed on tissue. Significantly more epithelial cells were positive for NOS2 in CD than in UC (Figure 4; *P* = 0.0017) while no significant difference was observed comparing stromal cells of CD and UC. Increased levels of epithelial NOS2 in CD were consistent with an increase in senescence-associated HP1γ in CD compared to UC (Figure 1A). We were not able to stratify CD tissues to investigate if epithelial cell NOS2 expression correlated with senescence because all CD tissues had high senescence, possibly due to the combined effects of NO• from macrophages and intracellular NO• from epithelial cells. There are no primary epithelial cells of a colonic origin to test our proposal that NO• from epithelial cells is directly related to senescence *in vitro*. We introduce the hypothesis that intracellular (epithelial) NO• may be involved in senescence in CD, and this may be tested should appropriate model systems become available.

### MicroRNAs are Associated with NOS2 and Senescence

**Figure 5:**
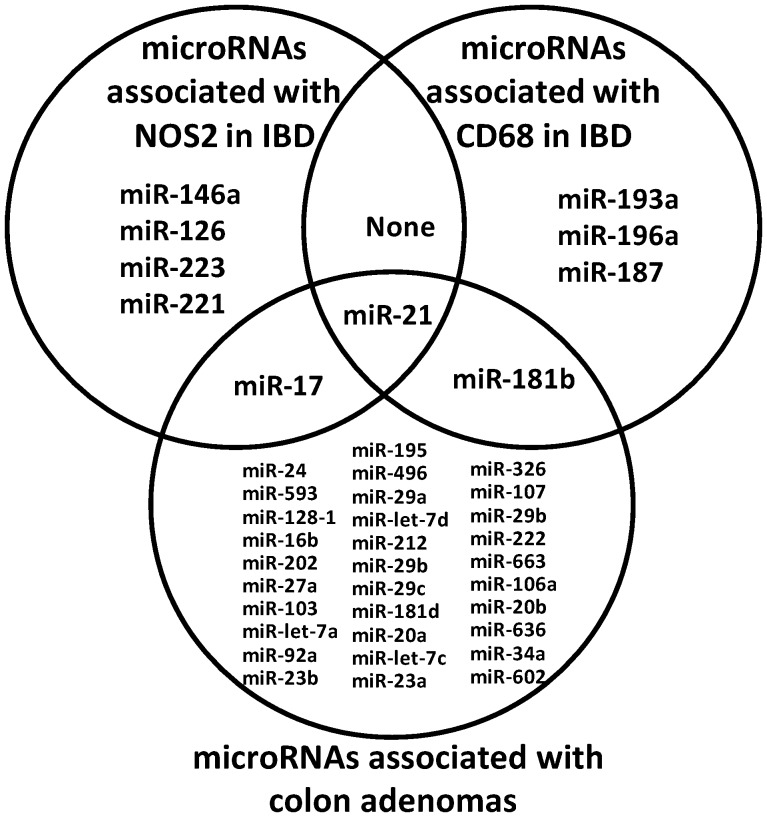
Association of microRNAs with NOS2 and CD68 expression in IBD and microRNAs altered in colon adenomas. The Venn diagram displays microRNAs that were significantly associated with the mRNA expression of NOS2 (*P*<0.001) and CD68 (*P*<0.05) and those microRNAs that are altered in colon adenomas (*P*<0.001) based on microRNA microarray profiling. MiR-21 was found to be associated in all three comparisons suggesting a potential role for this microRNA in senescence.

After establishing that macrophages are associated with senescence in UC, and directly induce senescence in an NO•-dependent fashion *in vitro*, we performed microRNA microarray expression analysis on RNA extracted from both UC and CD tissues to identify candidate microRNAs which may have a role in senescence. We measured the expression of NOS2 and the macrophage marker, CD68 by qRT-PCR and analyzed associations between these and microRNA expression levels. We identified 6 microRNAs (miR-21, miR-17, miR-146a, miR-126, miR-223 and miR-221) that were associated with NOS2 expression (*P*<0.001, FDR <5%) indicating that these microRNAs are potentially involved in NO• associated senescence (Figure 5, Table S2). While no microRNAs were associated with CD68 expression at the stringent statistical cutoff of *P*<0.001, a more lenient cutoff identified 5 microRNAs that were associated with CD68 (*P*<0.05), including miR-21, providing evidence that miR-21 may be involved in both macrophage and NOS2 induced senescence.

Colon adenomas are premalignant lesions in which high levels of cellular senescence serves as a barrier to a malignant transformation [12], [38]. In order to identify microRNAs whose expression is associated with cellular senescence in multiple disease states, we examined microRNAs expression patterns in senescent adenomas to compare to senescent-associated microRNAs from UC and CD. As expected, adenomas expressed high levels of senescence-associated HP1γ (Figure S3E) and we previously have shown that these adenomas are positive for SA-βgal [39]. This confirms high levels of cellular senescence in these tissues. We next performed microRNA microarray profiling of colonic adenomas and paired normal tissue, and compared these results with our findings in IBD. Among the 31 microRNAs altered in adenomas (Figure 5, Table S3), miR-21 had the highest fold change increase in adenomas, consistent with our previous qRT-PCR data on miR-21 in adenomas [40]. MiR-21 was the only microRNA that was associated with both NOS2 and CD68 in IBD; thus miR-21 is commonly associated with macrophages linked to senescence in IBD and *in vitro*, and NO• which induces senescence *in vitro*. We have previously reported that miR-21 expression is associated with NOS2 expression in colon cancer [41] providing more confidence that this association is relevant. This suggests a potential role for this microRNA in NO• and inflammation-associated senescence, and future investigations will focus on the possible role of miR-21 *in vivo*, and mechanistic experiments *in vitro* to show direct effects that cannot be tested in human tissue. Interestingly, miR-17 was commonly altered in adenomas and associated with NOS2 in IBD while miR-181b was altered in adenomas and associated with CD68.

### Conclusions

Cellular senescence is one of the many links between aging and cancer, and may occur through several mechanisms including telomere dysfunction and oncogenic stress [42]. UC has been theorized to be a disease of cellular aging, based on evidence of telomere attrition and chromosomal instability [10], [43]. We found that senescence-associated HP1γ expression in colonic epithelia was increased in UC colons in association with a high number of macrophages. This association is consistent with the hypothesis that macrophages may directly or indirectly induce cellular senescence in adjacent epithelial cells, which we observed *in vitro*. Our findings suggest that in addition to cell intrinsic mechanisms such as replicative telomere shortening, microenvironmental cues such as infiltrating immune cells and their derived factors may regulate epithelial cell senescence in cancer-prone lesions. This is consistent with a recent report associating high levels of infiltrating lymphocytes with telomere shortening and senescence in UC [44]. Stromal senescent fibroblasts can also secrete proinflammatory cytokines, e.g., IL-6, IL-8 and Gro-α [45] that can contribute to IBD, consistent with our observations.

High macrophage infiltration was associated with increases in the DDR sensor molecule γH2A.X, an indicator of active DNA damage response signaling by upstream DDR kinases including ATM and ATR [46], [47], and phosphorylation of downstream stress response protein Chk2 in colonic epithelial cells of inflamed, cancer-prone tissue of UC patients. The increased level of γ-H2A.X in UC colon, when compared to normal colon, is consistent with a previous report [10] and suggests that DDR may lead to cellular senescence in a proinflammatory environment. It is not clear if the DDR response associated with macrophages *in vivo*, and induced by NO• *in vitro,* is pro- or anti-carcinogenic, but DDR has previously been hypothesized to be an anti-cancer barrier [11]. It is possible that macrophages and/or NO• induce the DDR pathway leading to cellular senescence, and limiting proliferation of cells as a barrier to cancer. Alternatively, senescent cells in the microenvironment may themselves be procarcinogenic by secreting cytokines including IL-6, IL-8, IL-1α and IL-1β [38], [48].

Our *in vitro* data suggest that macrophages induce cellular senescence in a NO• dependent manner. Macrophages or clinically relevant concentrations of NO• induce cellular senescence in normal human fibroblasts and the NO• synthase inhibitor L-NAME proportionally reduced both NO• and senescence. L-NAME is often considered a nonselective NO• synthase inhibitor, but it has been previously shown to more efficiently block NO• production from NOS3. NOS3 is known to be important in the regulation of NOS2 expression [49], thus we hypothesize that L-NAME may decrease the amount of NO• by inhibiting NOS3 activity and down regulating NOS2 expression. This may be especially relevant at the low levels of steady state NO• (50–100 nmol) expected with 10 µM of Sper/NO• [50]. NO• has been implicated in the activation of the DDR pathway in cell lines and primary cells of patients with Barrett’s esophagus. Specifically, NO• donor MAHMA-NONOate induces γH2A.X in Barrett’s esophagus non-dysplastic, high-grade dysplastic, and adenocarcinoma cell lines [51]. Interestingly, Dickey *et al*. have shown that NO• induces γH2A.X *in vitro*, and that γH2A.X is induced in unexposed cells adjacent to cells exposed to irradiation [52]. We have also shown that NO• induces γH2A.X in normal human fibroblasts.

NOS2 is increased in colon adenomas [8]; when NOS2 is overexpressed in p53 wild type cells, p53 accumulates and induces a negative feedback loop that down regulates NOS2 expression to decrease nitrosative stress [53]. In contrast, NOS2 overexpression of NOS2 in p53 mutant cells leads to increased angiogenesis and tumorigenicity of human cancer cells as xenografts in immunosuppressed mice [54]. We hypothesize that NOS2 expression in IBD patients with intact and activated p53 serves as a barrier to carcinogenesis, based on the literature and our *in vitro* data that NO• induces senescence and DDR. However, once p53 is inactivated in IBD by mutation [5], nitrosative stress induced by NOS2 may not induce senescence due to loss of p53, and may become procarcinogenic. We plan to investigate these hypotheses should *in vitro* models with primary epithelial cells lines become available.

The miR-146a/b family of microRNAs that are elevated in senescent fibroblasts and thought to modulate senescence through effects on IL-6 and IL-8 [55]. We find that miR-146 expression correlates to NOS2 expression levels in IBD tissues, consistent with a role for miR-146 and NO• in senescence. MiR-21 is an oncogenic microRNA with known roles in inflammation, cell proliferation and tumorigenesis. We found that miR-21 expression is associated with high NOS2 and CD68 expression in UC and CD, as well as colon adenomas. Mir-21 has previously been shown to be increased in active ulcerative colitis [56] and upregulated during DNA damage by hydrogen peroxide and ionizing radiation associated with reactive oxygen species [57]. Inflammatory stimuli, such as *Corynebacterium parvum*-induced inflammation in mice, results in elevated levels of miR-21 [58]. MiR-21 can activate the NO• pathway *in vitro* [59] and miR-21 levels can be regulated by NF-kappaB [60]. Our data suggests that miR-21 may have a role in senescence, although future studies are needed to confirm these results in a second population using a more sensitive assay like RT-PCR, and in *in vitro* studies to show a direct effect. While at first a role in senescence may seem counterintuitive given the oncogenic role of miR-21, other oncogenes, including RAS [61], have roles in oncogene-induced senescence. Interestingly, the RAS pathway has been shown to increase miR-21 expression [62], and NO• can activate the RAS pathway [63]. Therefore, it is possible that in IBD, NO• leads to RAS activation and miR-21 transcription that is in part responsible for senescence in IBD. Future studies should explore if miR-21 is induced by NO• in a RAS-dependent manner and contributes to senescence.

Our study revealed a significance difference in senescence between CD and UC; higher HP1γ-associated senescence was observed in CD than in UC, and may reflect a critical difference between these two chronic inflammatory diseases. Genome wide association studies have shown thus far that some susceptibility loci are shared by both UC and CD, while others are solely associated with one but not the other disease [64]–[67]. For example, inflammatory pathways involving IL-23/IL-17 are both implicated in UC and CD, but *NOD2* is associated solely with CD. *NOD2* is required for tolerization of macrophages to bacterial peptides, including ligands for TLR2 and TLR4 [68]. Macrophages from CD Leu1007insC Nod2 homozygote individuals fail to develop tolerance to repeated stimulation with ligands, leading to the production of TNFα, IL-1β, and IL-8 [68]. Mice carrying a similar variant of NOD2 have elevated levels of NF-kappaB and IL-1βin response to MDP [69]. TNFα and IL-1β both contribute to NOS2 expression and NO• production *in vivo* [35], and IL-8 has been shown to be a prosenescent cytokine important to senescence induced by DNA damage [48]. The presence of senescence cells can cause age-related, chronic conditions in addition to inhibiting carcinogenesis [70]. We summarize these data in a model (Figure S9), and propose that regulation of NO• by proinflammatory cytokines contributes to up regulation of the DNA damage response pathway and senescence based on our *in vitro* assays.

Our findings related to inflammatory bowel disease may be applicable to other precancerous states associated with inflammation, and also those associated with oncogenic stress. Macrophages have long been implicated in association with tumors [27], and many questions remain on how immunity is involved in carcinogenesis. Before now, there had been no direct connection established between macrophages or NO• and senescence. Future studies may focus on the modulation of senescence through immune response to improve cancer outcome.

## Supporting Information

Figure S1 **Antibodies against phospho-Chk2 (Thr68) and Chk2 are specific.** HCT116 Chk2−/− and parental Chk2+/+ isogenic cell lines (generously given by the Vogelstein Laboratory) growing in log phase were exposed to 12 Gy of ionizing radiation to induce phospho-Chk2, and harvested 1 hour later. Lysates from Chk2+/+ cells (0 Gy; lane 1, 12 Gy; lane 2), and lysates from Chk2−/− cells (0 Gy; lane 3, 12 Gy; lane 4) are indicated by numbers below each immunoblot. Antibody for (A) phospho-Chk2 (Thr68) used for immunohistochemistry, was determined to be specific by immunoblot, as illustrated by the appropriate sized band detected in irradiated Chk+/+ cells only. (B) Specificity of the Chk2 (clone 273) antibody was confirmed, as shown by the darkest band detected in only Chk2+/+ cells, regardless of irradiation. (C) Additional total Chk2 antibodies (clone 270; Stressgen) and (D) ascites from clone 273 (generously given by Jiri Bartek) were tested to confirm the results. Immunocytochemistry was also performed with (E) phospho-Chk2 (Thr68) and (F) Chk2 (clone 273) antibodies, with similar results. (IR− = O Gy gamma-irradiation, IR+ = 12 Gy gamma-irradiation).
(TIF)

Figure S2 **Inflammatory bowel disease colons have increased macrophage infiltration in the lamina propria compared to normal colons.** Macrophages were identified with anti-CD68 immunohistochemistry and quantified by enumerating the number of positive brown cells in the lamina propria. Ulcerative colitis and Crohn’s disease colons had an increased number of macrophages compared to normal colons (ANOVA, *P = *0.02; Dunn’s *P*<0.05 for both comparisons). There was no significant difference in the number of macrophages between colons from ulcerative colitis and Crohn’s disease patients.
(TIF)

Figure S3 **Senescent cells are detectable by both immunohistochemistry for HP1γ and enzyme activity for senescence associated β-galactosidase in inflammatory bowel disease.** A) A representative picture of senescence associated β-galactosidase positivity is shown in frozen sections from ulcerative colitis colon. Colonic epithelial cells showed distinct cytoplasmic blue staining at 100× and B) 400× magnification. (C) Cells of the lamina propria, adjacent to epithelial cells, also stained blue for SAβ-gal activity at 100× and (D) 400× magnification. E) A representative picture of colon adenoma tissue stained for HP1γ.
(TIF)

Figure S4 **Examples of immunohistochemistry for DNA damage response and p53-stress response markers.** Examples from inflammatory bowel disease colon sections were chosen to emphasize differences reflected in cell counts (represented in Figure 2). Positive cells are indicated by brown nuclear stain (DAB) and negative cells are shown with blue counterstaining (Hematoxylin). Positive staining for γH2A.X, phospho-Chk2, Chk2, p53, and p21 was nuclear. For normal tissues, areas with well-oriented crypts were available, and these are illustrated with the lumen oriented toward the top of the panel. A summary of this data is shown in Figure 2.
(TIF)

Figure S5 **Crohn’s disease colons show no difference in DNA damage or p53 activation in association with macrophage index.** Tissues from Crohn’s disease patients were evaluated by immunohistochemistry for γ-H2A.X, phospho-Chk2, total p53 and p21. Staining is not associated with low and high macrophage index (*P*>0.05).
(TIF)

Figure S6 **Macrophages and nitric oxide induce senescence in primary human fibroblasts.** Representative pictures are shown of positive (blue) and negative (white) cells, indicative of senescence-associated β-galactosidase **(**SA-βgal) enzyme activity. A) A low density of normal human fibroblasts (MRC5) were cocultured with macrophages (ANA-1) in 6-well plates at a ratio of 3∶1, respectively. Cocultures were allowed to grow for 7 days with and without the nitric oxide inhibitor L-NAME (500 µM). Macrophages induced cellular senescence in fibroblasts, as shown by the enlarged, blue, SA-βgal positive cells. L-NAME partially abrogated the induction of senescence in fibroblasts. Cells grown in media only were negative for SA-βgal. (B) Normal human fibroblasts were incubated with 10 µM, 3 µM, and 0.9 µM Spermine NONOate (Sper/NO•) over night (16 hrs). After treatment, the cells were fixed and stained for SA-βgal. Treatment with 10 µM and 3 µM Sper/NO• induced a significant number of enlarged, SA-βgal positive cells, when compared cells grown in media alone (negative control). Treatment with 0.9 µM Sper/NO• did not induce significant levels of SA-βgal positive cells. Hydrogen peroxide (positive control; 200 µM) induced SA-βgal activity.
(TIF)

Figure S7 **Steady state nitric oxide was highest at 381 nM at 4 hours, and nitric oxide was decayed by 6 hours.** The decay of Spermine NONOate (Sper/NO•) was determined by measuring steady state nitric oxide on a nitric oxide gas analyzer. A 100 µl aliquot of 100 µM of Sper/NO• in serum-free media was aspirated by gas-free syringe into the sampling chamber at 0, 0.75, 1.5, 2, 4, and 6 hour time points.
(TIF)

Figure S8 **Nitric oxide induces DNA damage response in primary human fibroblasts in culture.** Normal human fibroblasts (MRC5) were incubated with media alone (negative control) or 10 µM Spermine NONOate (donor) and assayed for γH2A.X foci by immunofluorescence as indicated by FITC (green) fluorescence. DAPI (purple blue) was used to identify nuclei, and this image was overlaid with FITC top create a composite. (A, C, E,) Cells grown in media alone were negative for γH2A.X.foci at 400× magnification. (G) Enlargement of a single cell treated with media alone (indicated by the red box in panel C) shows that there is very little FITC fluorescence for γH2A.X. (B, D, F) Cells treated with donor Sper/NO• became enlarged and failed to divide, leading to a low density of cells. Due to the low cell density, it was difficult to capture multiple cells in one 400× magnification field, thus each panel is a composite of four pictures of one single cell each. Each cell shows positive FITC fluorescence for γH2A.X foci. (H) Enlargement of a single cell treated with Sper/NO• (indicated by the red box in panel D) shows distinct focal fluorescence. Panels are shown at 400× magnification except for γH2A.X high magnification panels (G, H), which show an enlarged section (red rectangle) from the γH2A.X panels (C, D).
(TIF)

Figure S9 **Proposed model of DNA damage response and senescence resulting from a polymorphism in NOD2/CARD 15 carried by Crohn’s disease patients.** Previous studies have illustrated that a polymorphism in NOD2 carried by Crohn’s disease patients results in the loss of tolerization to bacterial peptide, including TLR2 and TLR4 ligands upon restimulation. [68] This may result in the production of NF-κB and proinflammatory cytokines that are part of a chronic inflammatory response. [69] Cytokines IL-1β and TNF-α can lead to the induction of NOS2 to secrete nitric oxide. [35] Our data suggest that nitric oxide may induce DNA damage and result in cellular senescence.
(TIF)

Table S1 **Characteristics of the study populations.** ^1^CHTN, Cooperative Human Tissue Network.
(XLS)

Table S2 **MicroRNAs are associated with NOS2 and CD68 expression in Ulcerative Colitis (UC) and Crohn's Disease (CD) tissues.** NOS2 and CD68 expression levels were dichotomized based on median expression levels. Class comparison analyses identified microRNAs that were differentially expressed when comparing high vs low expressing groups for NOS2 and CD68. FDR, False discovery rate.
(XLS)

Table S3 **MicroRNAs that are altered in colon adenomas compared to adjacent nonadenoma tissue.** Class comparison analyses identified microRNAs that were differentially expressed in colon adenomas. FDR, False discovery rate.
(XLS)

Materials and Methods S1 These are methods that describe the protocols for immunohistochemical anaylsis, coculture and cell culture studies, statistical analysis, senescence-associated β-galactosidase studies, nitric oxide quantification, immunofluorescence, RNA isolation, microRNA profiling and qRTPCR.
(DOC)
