# Supplementary figures and images for: Macrophages, Nitric Oxide and microRNAs Are Associated with DNA Damage Response Pathway and Senescence in Inflammatory Bowel Disease
Source: PLoS One. 2012 Sep 6;7(9):e44156. doi: 10.1371/journal.pone.0044156 (PMC3435404; doi:10.1371/journal.pone.0044156)

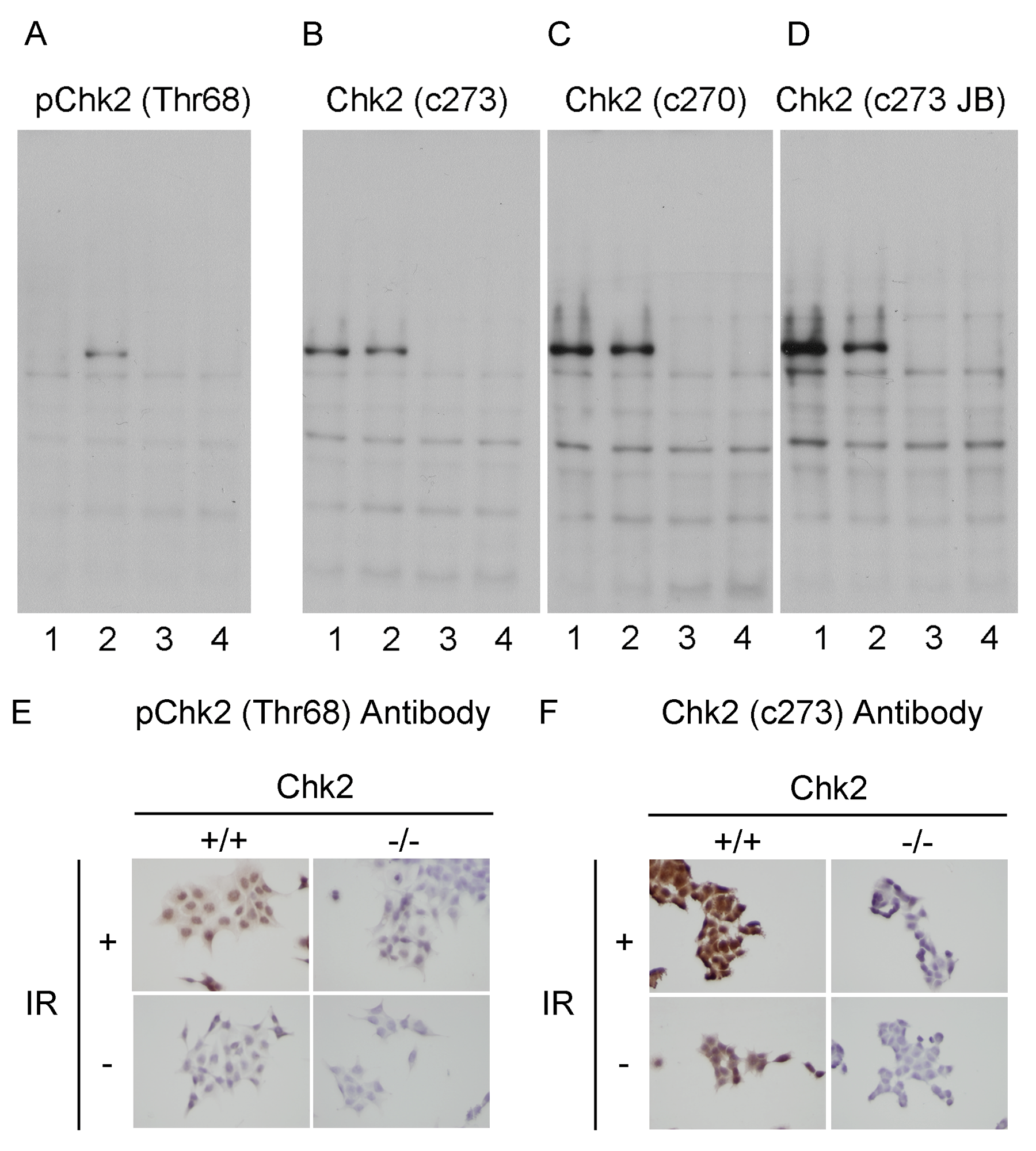

Supplement: Figure S1 — Antibodies against phospho-Chk2 (Thr68) and Chk2 are specific. HCT116 Chk2−/− and parental Chk2+/+ isogenic cell lines (generously given by the Vogelstein Laboratory) growing in log phase were exposed to 12 Gy of ionizing radiation to induce phospho-Chk2, and harvested 1 hour later. Lysates from Chk2+/+ cells (0 Gy; lane 1, 12 Gy; lane 2), and lysates from Chk2−/− cells (0 Gy; lane 3, 12 Gy; lane 4) are indicated by numbers below each immunoblot. Antibody for (A) phospho-Chk2 (Thr68) used for immunohistochemistry, was determined to be specific by immunoblot, as illustrated by the appropriate sized band detected in irradiated Chk+/+ cells only. (B) Specificity of the Chk2 (clone 273) antibody was confirmed, as shown by the darkest band detected in only Chk2+/+ cells, regardless of irradiation. (C) Additional total Chk2 antibodies (clone 270; Stressgen) and (D) ascites from clone 273 (generously given by Jiri Bartek) were tested to confirm the results. Immunocytochemistry was also performed with (E) phospho-Chk2 (Thr68) and (F) Chk2 (clone 273) antibodies, with similar results. (IR− = O Gy gamma-irradiation, IR+ = 12 Gy gamma-irradiation). (TIF) [file pone.0044156.s001.tif]

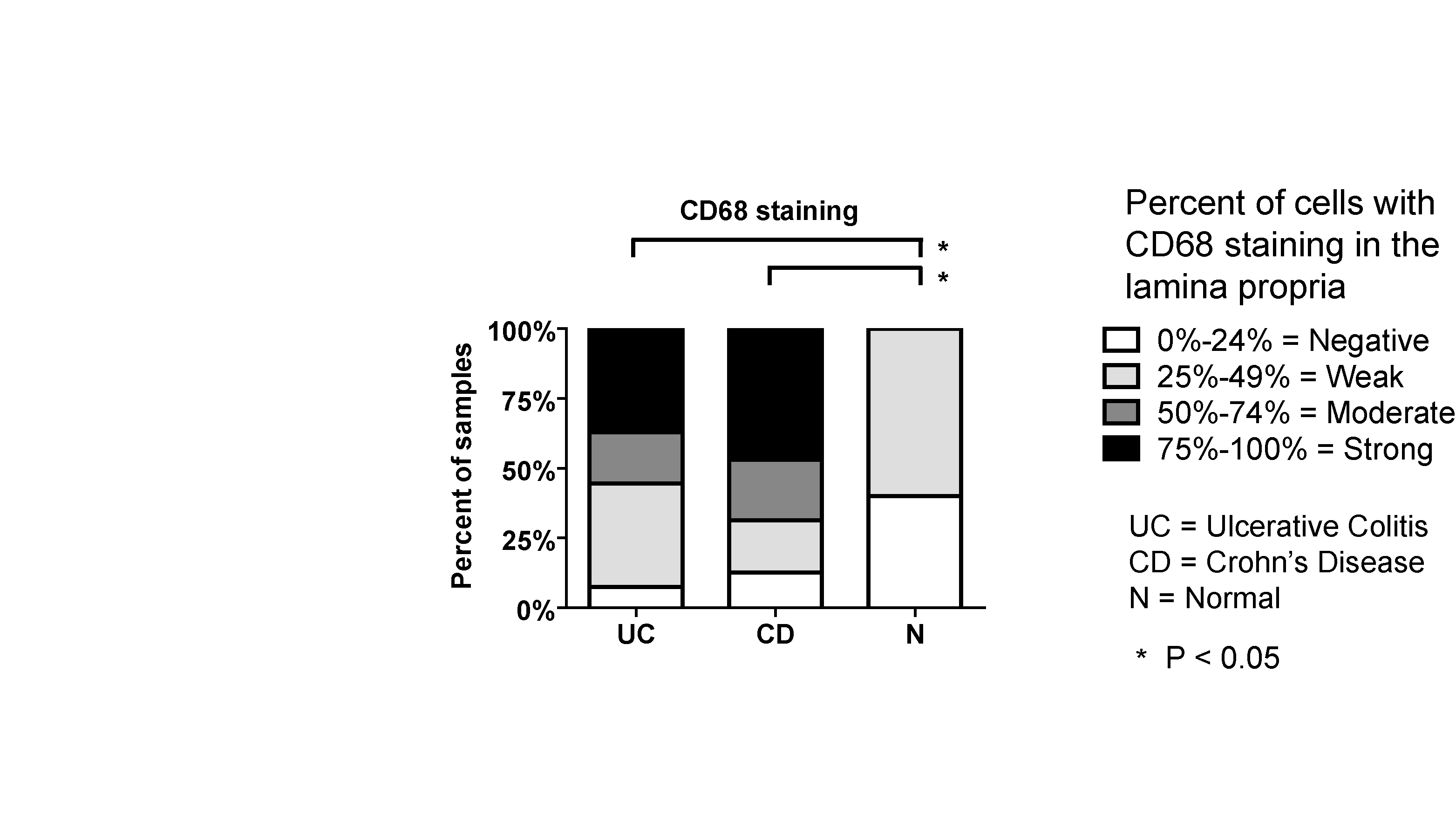

Supplement: Figure S2 — Inflammatory bowel disease colons have increased macrophage infiltration in the lamina propria compared to normal colons. Macrophages were identified with anti-CD68 immunohistochemistry and quantified by enumerating the number of positive brown cells in the lamina propria. Ulcerative colitis and Crohn’s disease colons had an increased number of macrophages compared to normal colons (ANOVA, P = 0.02; Dunn’s P<0.05 for both comparisons). There was no significant difference in the number of macrophages between colons from ulcerative colitis and Crohn’s disease patients. (TIF) [file pone.0044156.s002.tif]

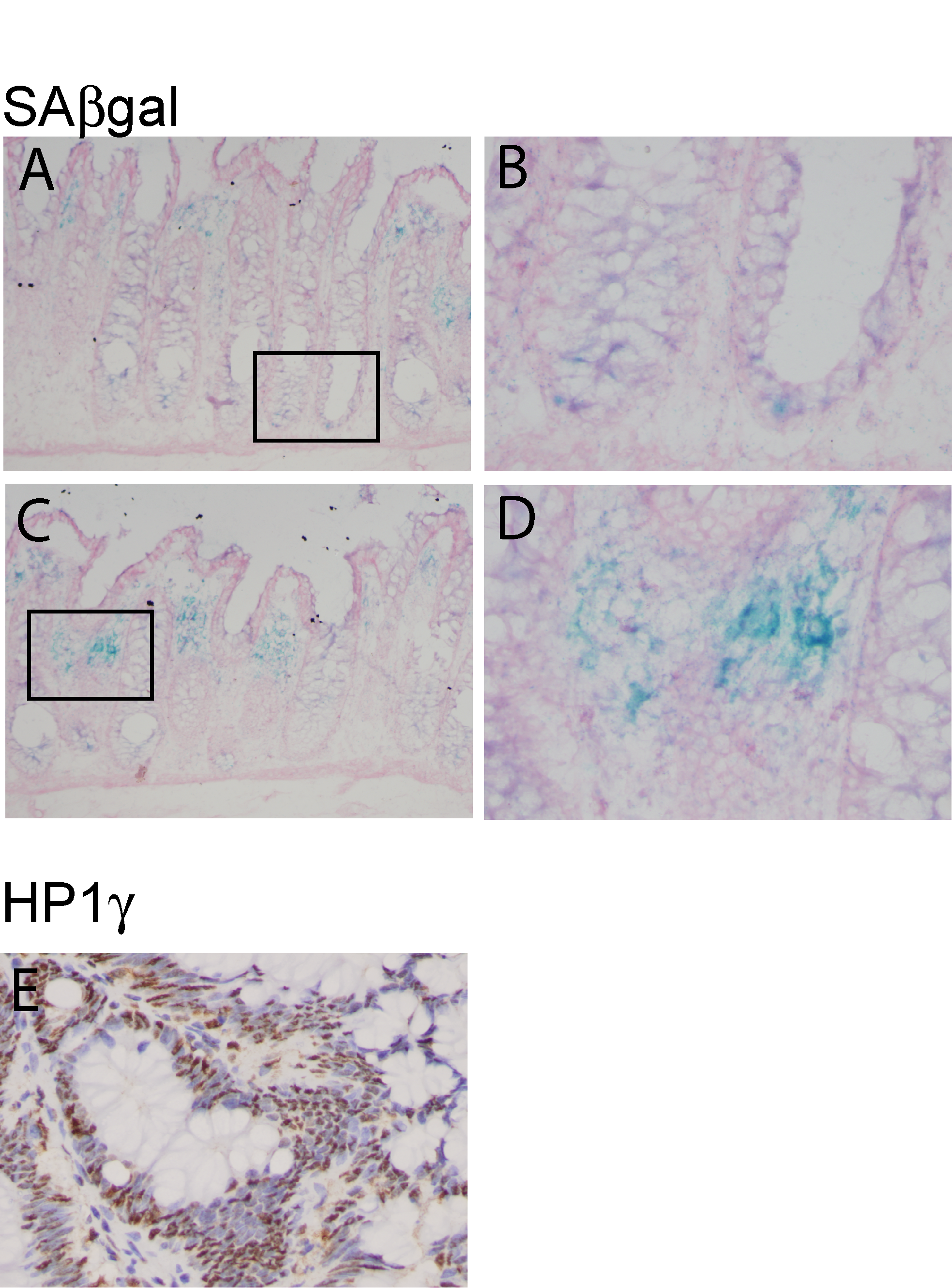

Supplement: Figure S3 — Senescent cells are detectable by both immunohistochemistry for HP1γ and enzyme activity for senescence associated β-galactosidase in inflammatory bowel disease. A) A representative picture of senescence associated β-galactosidase positivity is shown in frozen sections from ulcerative colitis colon. Colonic epithelial cells showed distinct cytoplasmic blue staining at 100× and B) 400× magnification. (C) Cells of the lamina propria, adjacent to epithelial cells, also stained blue for SAβ-gal activity at 100× and (D) 400× magnification. E) A representative picture of colon adenoma tissue stained for HP1γ. (TIF) [file pone.0044156.s003.tif]

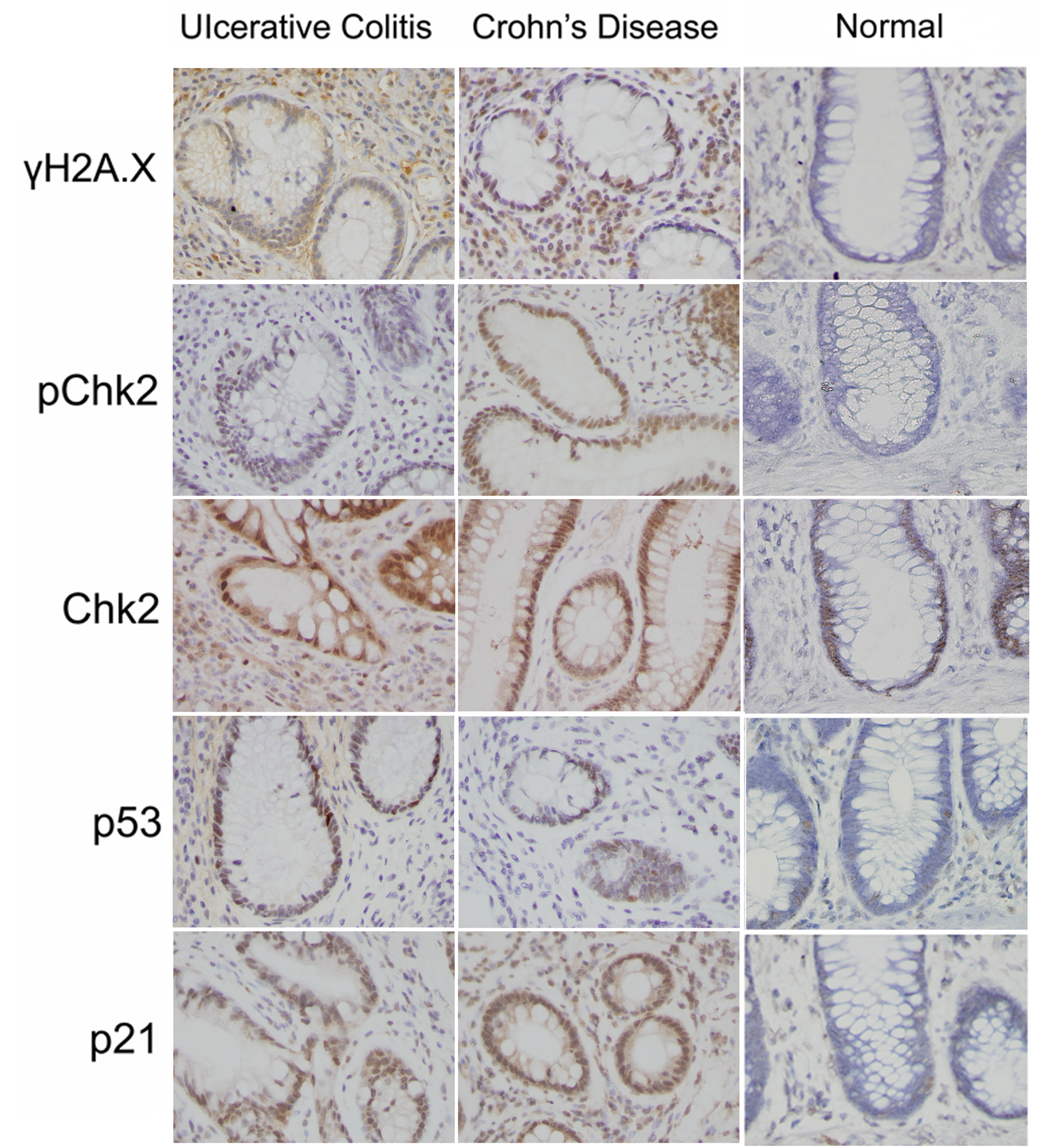

Supplement: Figure S4 — Examples of immunohistochemistry for DNA damage response and p53-stress response markers. Examples from inflammatory bowel disease colon sections were chosen to emphasize differences reflected in cell counts (represented in Figure 2). Positive cells are indicated by brown nuclear stain (DAB) and negative cells are shown with blue counterstaining (Hematoxylin). Positive staining for γH2A.X, phospho-Chk2, Chk2, p53, and p21 was nuclear. For normal tissues, areas with well-oriented crypts were available, and these are illustrated with the lumen oriented toward the top of the panel. A summary of this data is shown in Figure 2. (TIF) [file pone.0044156.s004.tif]

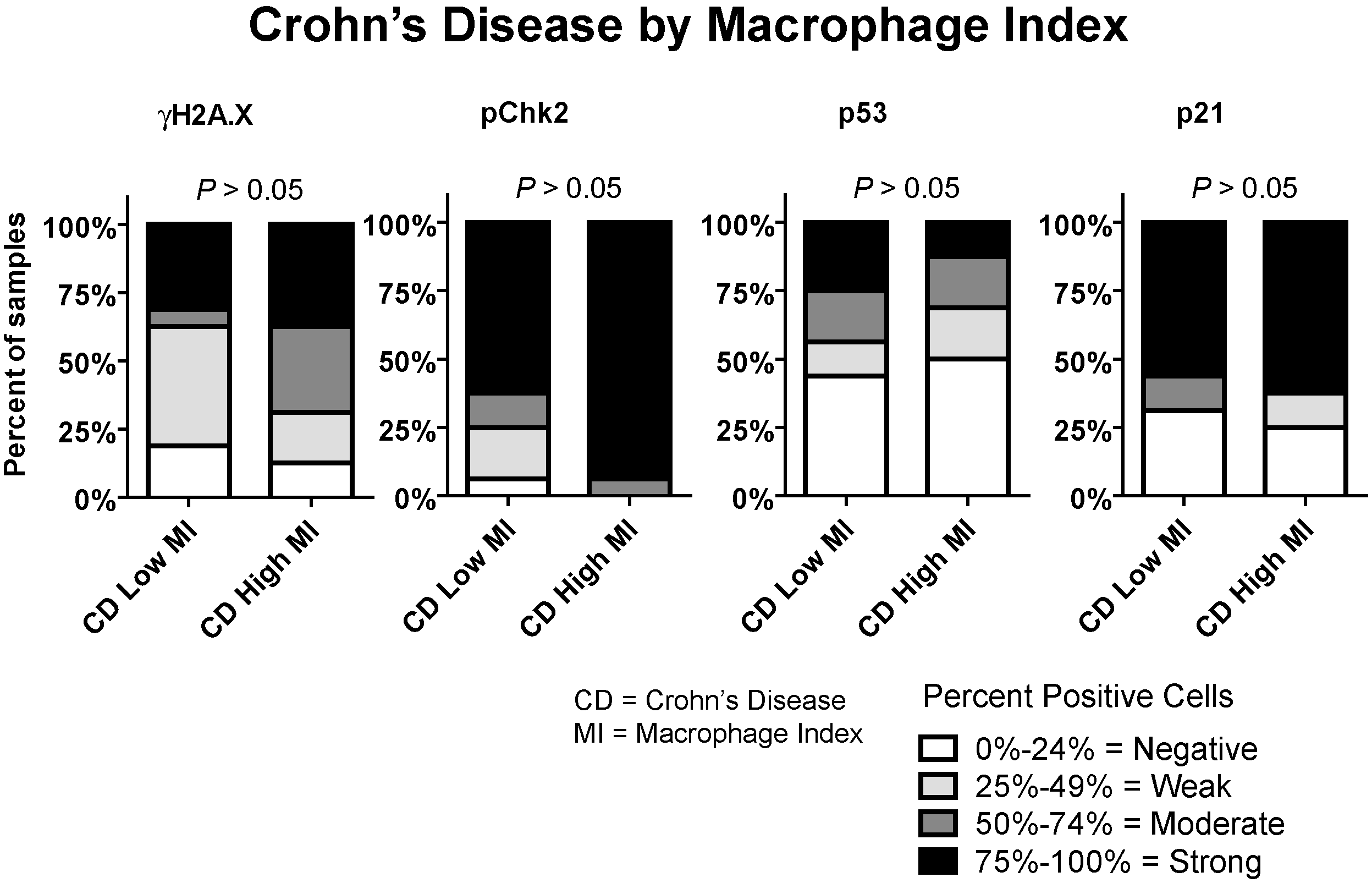

Supplement: Figure S5 — Crohn’s disease colons show no difference in DNA damage or p53 activation in association with macrophage index. Tissues from Crohn’s disease patients were evaluated by immunohistochemistry for γ-H2A.X, phospho-Chk2, total p53 and p21. Staining is not associated with low and high macrophage index (P>0.05). (TIF) [file pone.0044156.s005.tif]

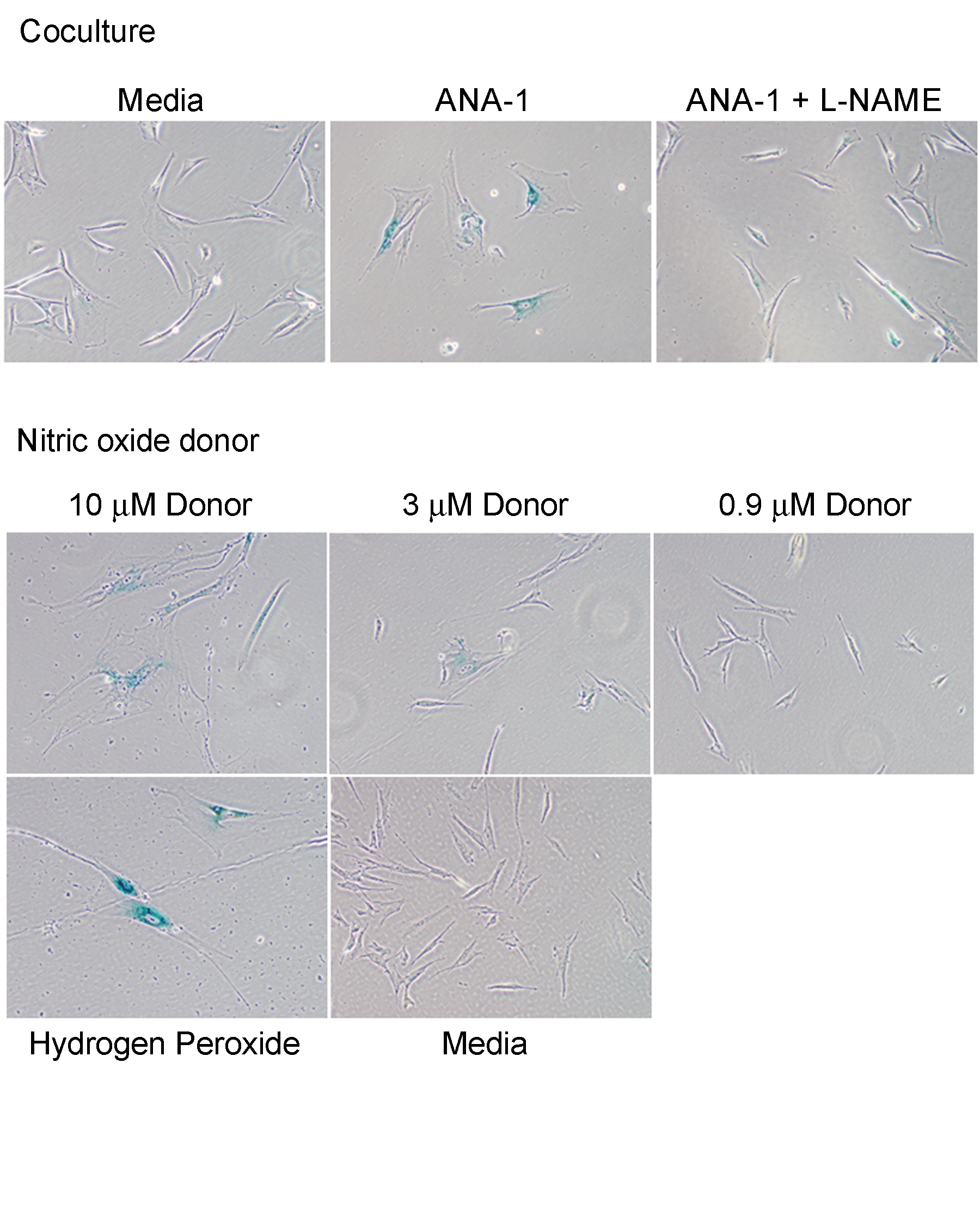

Supplement: Figure S6 — Macrophages and nitric oxide induce senescence in primary human fibroblasts. Representative pictures are shown of positive (blue) and negative (white) cells, indicative of senescence-associated β-galactosidase (SA-βgal) enzyme activity. A) A low density of normal human fibroblasts (MRC5) were cocultured with macrophages (ANA-1) in 6-well plates at a ratio of 3∶1, respectively. Cocultures were allowed to grow for 7 days with and without the nitric oxide inhibitor L-NAME (500 µM). Macrophages induced cellular senescence in fibroblasts, as shown by the enlarged, blue, SA-βgal positive cells. L-NAME partially abrogated the induction of senescence in fibroblasts. Cells grown in media only were negative for SA-βgal. (B) Normal human fibroblasts were incubated with 10 µM, 3 µM, and 0.9 µM Spermine NONOate (Sper/NO•) over night (16 hrs). After treatment, the cells were fixed and stained for SA-βgal. Treatment with 10 µM and 3 µM Sper/NO• induced a significant number of enlarged, SA-βgal positive cells, when compared cells grown in media alone (negative control). Treatment with 0.9 µM Sper/NO• did not induce significant levels of SA-βgal positive cells. Hydrogen peroxide (positive control; 200 µM) induced SA-βgal activity. (TIF) [file pone.0044156.s006.tif]

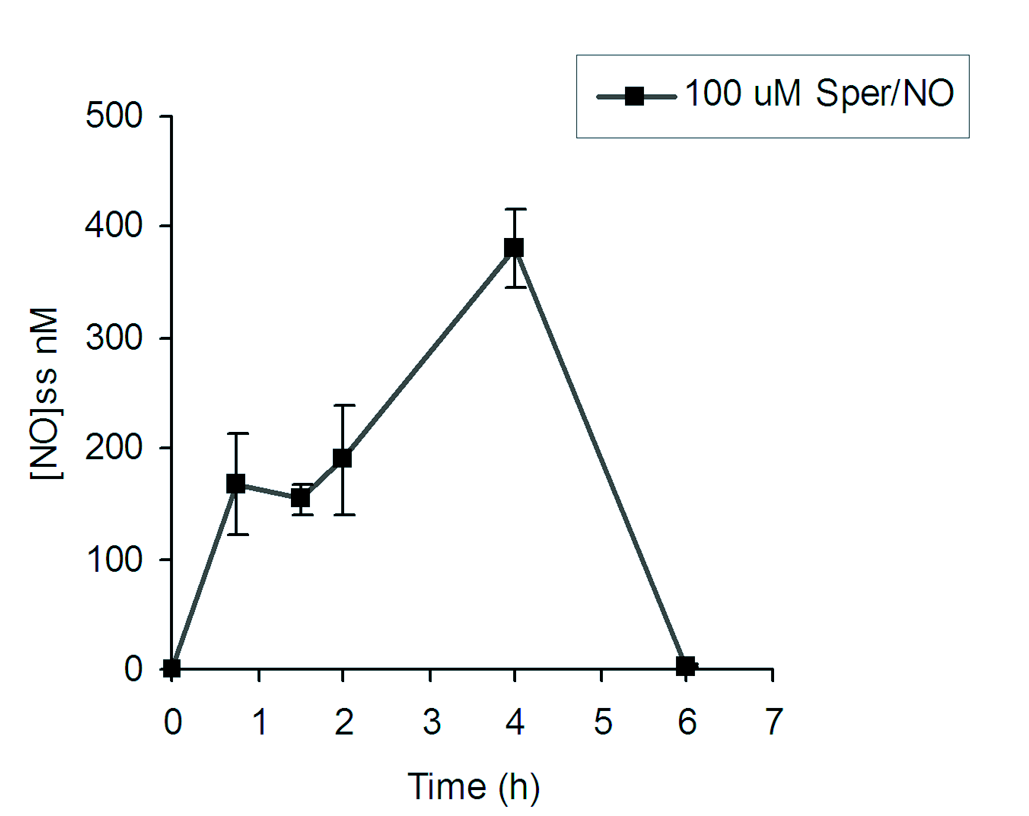

Supplement: Figure S7 — Steady state nitric oxide was highest at 381 nM at 4 hours, and nitric oxide was decayed by 6 hours. The decay of Spermine NONOate (Sper/NO•) was determined by measuring steady state nitric oxide on a nitric oxide gas analyzer. A 100 µl aliquot of 100 µM of Sper/NO• in serum-free media was aspirated by gas-free syringe into the sampling chamber at 0, 0.75, 1.5, 2, 4, and 6 hour time points. (TIF) [file pone.0044156.s007.tif]

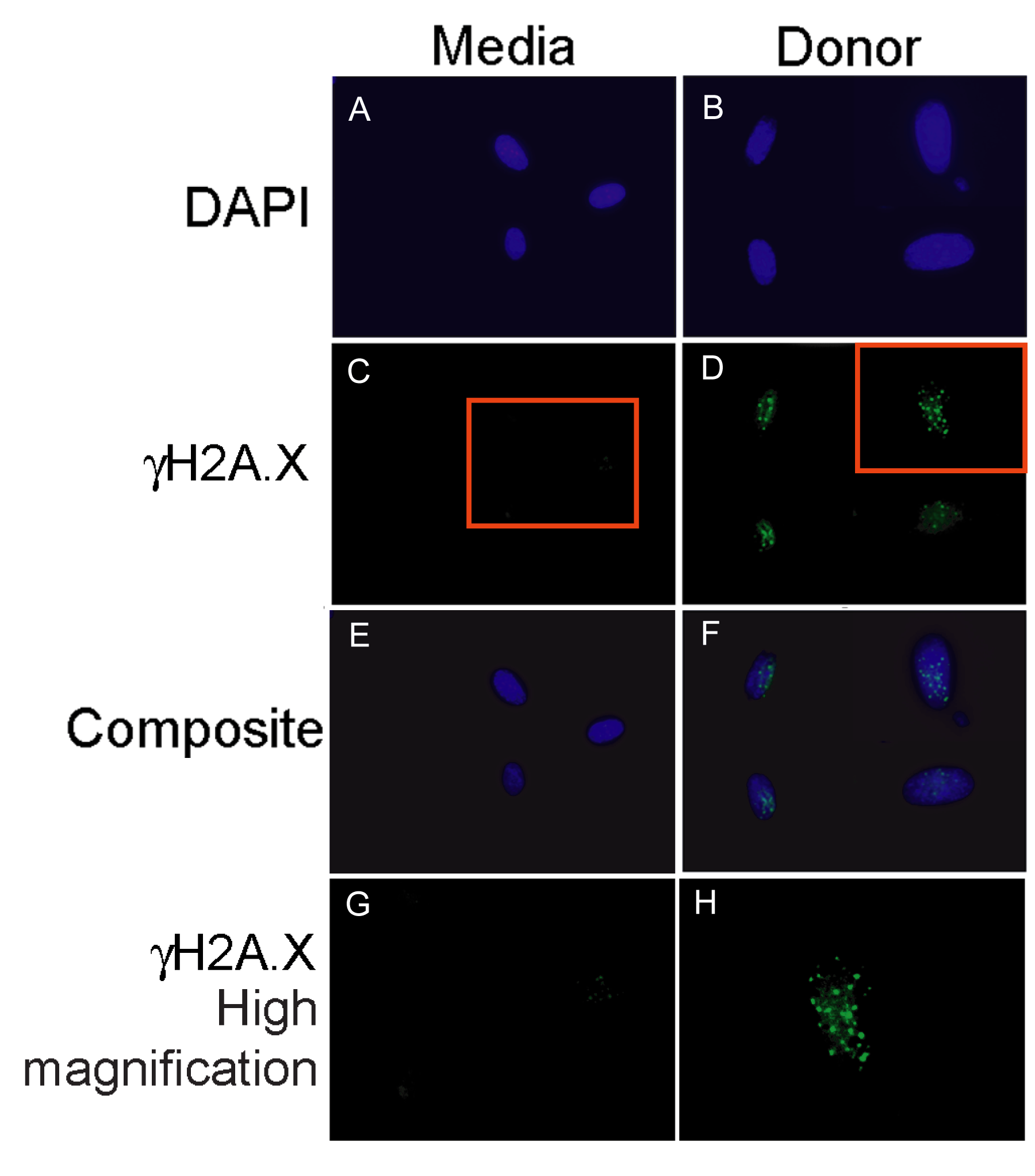

Supplement: Figure S8 — Nitric oxide induces DNA damage response in primary human fibroblasts in culture. Normal human fibroblasts (MRC5) were incubated with media alone (negative control) or 10 µM Spermine NONOate (donor) and assayed for γH2A.X foci by immunofluorescence as indicated by FITC (green) fluorescence. DAPI (purple blue) was used to identify nuclei, and this image was overlaid with FITC top create a composite. (A, C, E,) Cells grown in media alone were negative for γH2A.X.foci at 400× magnification. (G) Enlargement of a single cell treated with media alone (indicated by the red box in panel C) shows that there is very little FITC fluorescence for γH2A.X. (B, D, F) Cells treated with donor Sper/NO• became enlarged and failed to divide, leading to a low density of cells. Due to the low cell density, it was difficult to capture multiple cells in one 400× magnification field, thus each panel is a composite of four pictures of one single cell each. Each cell shows positive FITC fluorescence for γH2A.X foci. (H) Enlargement of a single cell treated with Sper/NO• (indicated by the red box in panel D) shows distinct focal fluorescence. Panels are shown at 400× magnification except for γH2A.X high magnification panels (G, H), which show an enlarged section (red rectangle) from the γH2A.X panels (C, D). (TIF) [file pone.0044156.s008.tif]

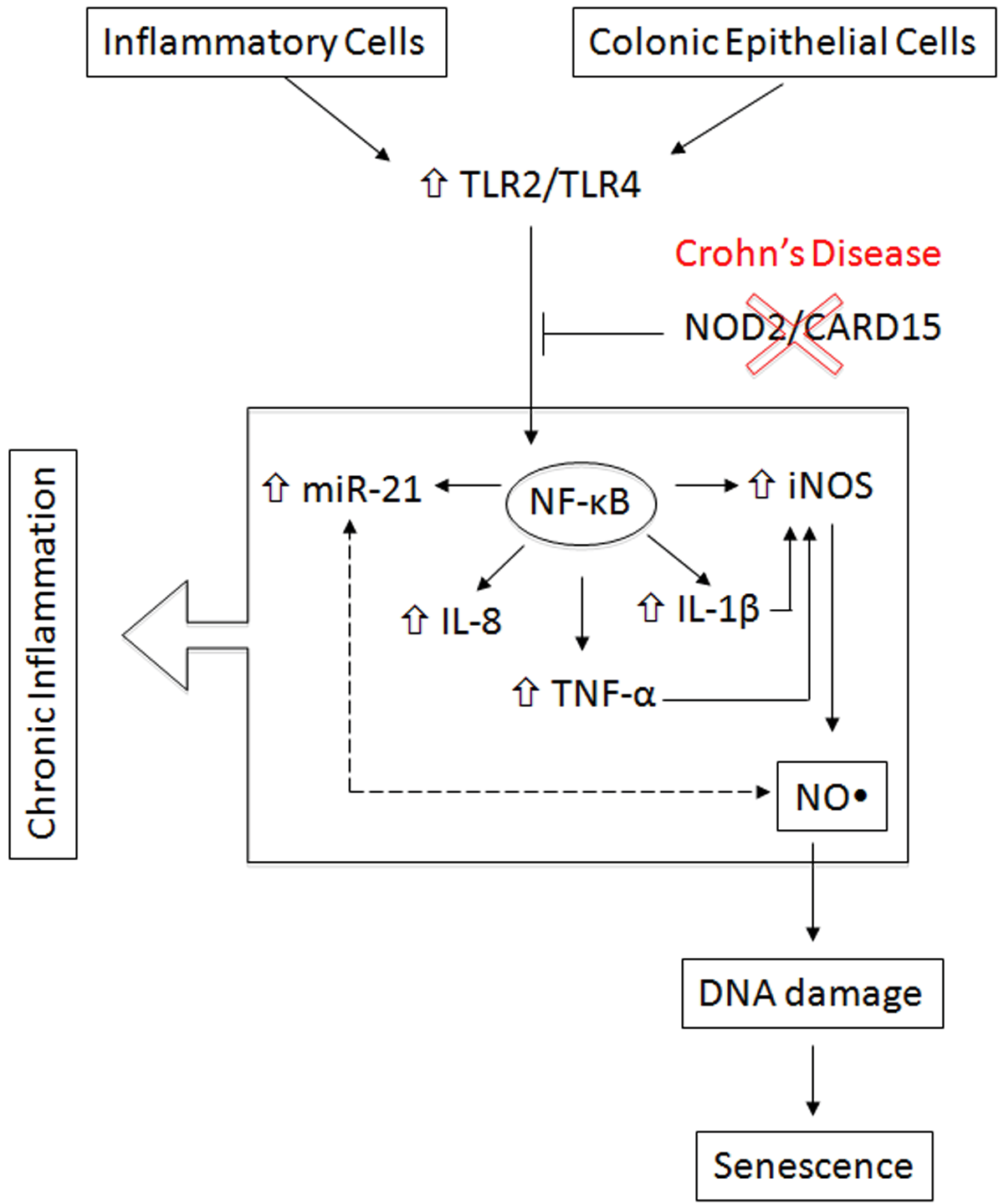

Supplement: Figure S9 — Proposed model of DNA damage response and senescence resulting from a polymorphism in NOD2/CARD 15 carried by Crohn’s disease patients. Previous studies have illustrated that a polymorphism in NOD2 carried by Crohn’s disease patients results in the loss of tolerization to bacterial peptide, including TLR2 and TLR4 ligands upon restimulation. [68] This may result in the production of NF-κB and proinflammatory cytokines that are part of a chronic inflammatory response. [69] Cytokines IL-1β and TNF-α can lead to the induction of NOS2 to secrete nitric oxide. [35] Our data suggest that nitric oxide may induce DNA damage and result in cellular senescence. (TIF) [file pone.0044156.s009.tif]
