# Supplementary material for: Macrophages, Nitric Oxide and microRNAs Are Associated with DNA Damage Response Pathway and Senescence in Inflammatory Bowel Disease
Source: PLoS One. 2012 Sep 6;7(9):e44156. doi: 10.1371/journal.pone.0044156 (PMC3435404; doi:10.1371/journal.pone.0044156)
Supplement: Materials and Methods S1 — These are methods that describe the protocols for immunohistochemical anaylsis, coculture and cell culture studies, statistical analysis, senescence-associated β-galactosidase studies, nitric oxide quantification, immunofluorescence, RNA isolation, microRNA profiling and qRTPCR. (DOC) [file pone.0044156.s013.doc]

**Supplemental Materials & Methods.**

**Antibodies for immunohistochemistry**. Five-micron serial sections from formalin-fixed, paraffin-embedded (FFPE) tissues were used for IHC. Identification of DDR activation was performed by IHC for γH2A.X (phospho-histone H2A.X Ser139) using a monoclonal mouse anti-human antibody (JBW103; Upstate, Lake Placid NY). The following antibodies were used to evaluate activation of the p53-network: polyclonal rabbit anti-human phospho-Chk2 (Thr68; 2661; Cell Signaling, Danvers, MA), monoclonal mouse anti-human Chk2 (clone 273, KAM-CC113; Stressgen, Victoria, BC, Canada), monoclonal mouse anti-human p53 (DO7; DAKO, Carpinteria, CA), and mouse monoclonal anti-human p21 (CP74; Neomarkers, Freemont, CA). One slide for γH2A.X was excluded due to uneven staining. Mouse monoclonal antibody against senescence-associated HP1γ (MAB3450; Chemicon, Billerica, MA) was used to quantify senescence [1,2]. Immunohistochemistry for NOS2 was performed with monoclonal mouse anti-NOS2 antibody (610329; BD Transduction, San Jose, CA).

All primary antibodies were detected using corresponding secondary antibodies from anti-mouse or anti-rabbit Envision+ (DAKO, Carpinteria, CA) or Vectastain Elite (Vector Labs, Burlingame, CA) kits. Blocking peptide against polyclonal antibody for phospho-Chk2 (Cell Signaling, Danvers, MA) was used in competition assays to confirm specificity of IHC, as reported by the manufacturer, to confirm antibody specificity. Further, phospho-Chk2 (Thr68; 2661; Cell Signaling) and total Chk2 (clone 273, KAM-CC113) antibodies were shown to be specific by performing immunoblots on cell lysates and immunocytochemistry on cells from HCT116 Chk2 -/- and parental HCT116 Chk2 proficient colorectal cell lines generously given by the Vogelstein Laboratory [3]. Briefly, cell lines were exposed to 12 Gy ionizing radiation and harvested one hour later to induce Chk2 phosphorylation. Additional total Chk2 antibodies (clone 270, KAM-CC112; Stressgen, Victoria, BC, Canada) and ascites from clone 273 (generously given by Jiri Bartek) were tested by immunoblot for specificity (Figure S1). IHC for Chk2 has previously been reported in human colon as being positive in almost all epithelial cells [4]. Our experiments showed that IBD tissues were similar, with all sections 75-100% positive (Figure 2).

Tissue sections were stained for CD68, a marker of macrophages and monocytes [5]. Mouse monoclonal antibody against human CD68 was incubated with tissue sections at the manufacturer’s recommended concentration (KP-1; Neomarkers, Freemont, CA). Secondary antibody incubation and detection were performed with the anti-mouse Envision+ kit (DAKO, Carpinteria, CA).

**Immunohistochemical Anaylsis.** For HP1γ, a combined score of intensity and distribution was used to score staining (A. G.; [6]) to reflect the marked differences in both intensity and number of positive cells between UC and CD, which was not observed for other markers. Intensity was scored from 0-3, representing negative, weak, moderate, or strong intensity, respectively. Distribution was scored from 0-4, with zero for <10%, one for 10-29%, two for 30-49%, three for 50-79%, and four for 80% or greater. Scores for intensity and distribution were added, and divided into 4 groups for a cumulative score: 1) 0-1; 2) 2-3; 3) 4-5; 4) 6-7.

**Coculture and cell culture treatment.** Normal human fibroblast strains MRC-5, and WI-38 were obtained from the Coriell Institute for Medical Research (Camden, NJ). Murine macrophage strain ANA-1 were a gift from G. Cox [7]. Cells were grown in DMEM media without phenol red. Media were supplemented with 10% FBS (Biofluids, Rockville, MD), 4 mM glutamine (Biofluids), penicillin (10 units/ml), and streptomycin (10 μg/ml, Biofluids).

Cocultures were established by seeding 2500 normal human fibroblasts per well in a 6-well dish with 2 mL of media, and 833 macrophages to establish an initial 1:3 ratio of fibroblasts to macrophages. Cultures were allowed to grow for 7 days, and 200 μL of media was removed and replaced each day to replenish media contents. Coculture experiments for senescence were repeated three times, with three technical replicates for each experiment. A minimum of 1500 cells were evaluated for senescence in each replicate. Cultures were not allowed to reach confluence because confluent cells may stain blue when evaluated by the SA-βgal assay. Macrophages were shaken off before evaluation of senescence in fibroblasts.

Spermine NONOate (Sper/NO•; Sigma) was used as a nitric oxide donor to test for the induction of senescence in normal human fibroblasts in three separate experiments, with triplicate wells for each repetition. At least 1500 cells were evaluated for senescence in each repetition.

**Statistical Analysis for IHC and cell culture.** Differences for IHC staining were compared by a nonparametric Mann-Whitney test for 2 samples, and by nonparametric ANOVA test (Kruskal-Wallis test) for more than 2 samples (e.g. ulcerative colitis versus Crohn’s disease versus normal). P < 0.05 was considered significant. If the ANOVA test was significant, pairs were compared by Dunn’s test and this value is reported. Pearson correlation coefficients were determined for comparisons of trends. A Chi-squared test was used if all samples in one group had no staining (i.e. pChk2 staining in normal samples).

Cell counts from SA β-gal in vitro experiments were analyzed by one-way ANOVA test for more than 2 samples, and P > 0.05 was considered significant. If the ANOVA test was significant, data were compared by Dunnett’s multiple comparison test, using untreated cells as the reference group. Amount of diffused nitric oxide measured by DAF fluorescence between cocultures with and without L-NAME was analyzed by paired, two-tailed t-test. Prism 5 was used (GraphPad, La Jolla, CA).

**Senescence-associated β-galactosidase.** Archival frozen IBD tissues were embedded in O.C.T. tissue embedding compound (Miles Scientific, Elkhart, IN), and stored at -80° C. Seven-micron, serial frozen sections were tested in triplicate for senescence-associated β-galactosidase (SA β-gal) immediately after cutting [8].

Positive controls included primary normal human fibroblasts (WI38, MRC5) grown in culture with Nutlin 3A, and previously published colon adenomas [9]. Normal human fibroblasts (WI38, MRC5) grown in coculture with macrophages (ANA-1) or exposed to Sper/NO• were washed 3 times with PBS, fixed with 4% paraformaldehyde for 5 minutes, and stained for SA-βgal activity at pH 6.0 as previously described [9]. Cultures were not allowed to reach confluence because confluent cells may stain blue when evaluated by the SA-βgal assay.

**Nitric Oxide Quantification.** The relative diffused nitric oxide in cultured cell media was quantified using DAF-FM diacetate (4-amino-5-methylamino-2’, 7’-difluorofluoroscein diacetate; DAF; Invitrogen). Media from 3 separate cultures was removed at 7 days and incubated with 5 μM DAF in 96 well, black wall clear bottom plates (Corning Inc., Corning, NY, USA). Each of the 3 sets of samples (media only, cococulture and coculture with L-NAME) was aliquoted sequentially to minimize the effect of the incubation with DAF. Fluorescence measurements were obtained on a Perkin Elmer HTS 7100 fluorescent plate reader (200 μl volume). Fluorescence measurements from cocultures were adjusted by subtracting the DAF fluorescence measured in media from wells with fibroblasts only.

The steady-state concentration of NO produced during Sper/NO• degradation was measured by NO gas analysis using a Seivers (Boulder, CO) NO gas analyzer. Aliquots of media (100 μl) from serum-free media containing 100 μM, 10 μM and 3 μM Sper/NO• were injected into the reaction chamber containing 0.5 mM NaOH to stop Sper/NO• decomposition as previously published [10].

**Immunofluorescence.** For detection of DNA damage response induction upon treatment with the nitric oxide donor Sper/NO•, immunofluorescence for γH2A.X was performed on MRC5 cells treated with 10 μM Sper/NO•. Cells were fixed in 2% paraformaldehyde

(PFA), 0.2% glutaraldehyde for 5 min, permeabilized in 0.1% Triton-X-100 for 5 min, and blocked for 1 h in normal donkey serum (Jackson ImmunoResearch, West Grove, PA). Cells were incubated with a 1:200 dilution monoclonal mouse anti-human γH2A.X antibody (JBW103; Upstate) overnight at 4º celcius and then washed and incubated with a 1:250 dilution of goat anti-mouse Alexa Fluor 594 for 1 h at room temperature. Cells were washed, dried, and coverslipped with DAPI. Images were visualized using a Zeiss Axioplan 2 flourescence microscope equipped with OpenLab 3.1.5 imaging software (Perkin Elmer, Waltham, MA). Filters for FITC and DAPI were used for visualization of Alexa Fluor 594 fluorochrome for γH2A.X and nuclear counterstaining, respectively. Two-color images were created on Photoshop 7.0 (Adobe Systems, San Jose, CA) by superimposing an image taken with a FITC filter, on top of a corresponding DAPI image at 50% opacity.

**RNA isolation, microRNA profiling, and qRTPCR:** RNA was isolated from flash frozen tissue using standard TRIZOL (Invitrogen, Carlsbad) methods, including 62 tissues from 31 CD patients, 54 tissues from 27 UC patients and 18 pairs of adenoma and adjacent uninvolved colon tissue. RNA was reverse transcribed using the cDNA Archive Kit (Applied Biosystems, Foster City, CA) with a 50 ng/μl final concentration. Taqman quantitative RT-PCR was used to measure expression levels of nitric oxide synthase 2A (NOS2A) (assay ID Hs00167257_m1) and cluster of differentiation 68 (CD68) (assay ID Hs00154355_m1) with 18s rRNA (assay ID Hs99999901_s1) as a normalization control. For quality control, any tissue that had 18s threshold cycle values >15 were considered poor quality and were removed. Expression of NOS2 and CD68 were classified as high or low based on median levels for UC and CD tissues separately. MicroRNA microarray profiling was performed as previously described^31^. Briefly, 5 μg of total RNA was labeled and hybridized to each microRNA microarray (Ohio State microRNA microarray version 2.0 for UC and CD tissues and Ohio State microRNA microarray version 4.0 for adenoma tissues.) Slides were scanned using a PerkinElmer ScanArray LX5K scanner. The data were preprocessed in R to remove probes with higher background intensities than foreground, remove probes with inconsistent measurements across the replicates, and to LOESS normalize the data. Date affect biases were removed using Partek Genomics Suite 6.5. Data was then imported into BRB array tools 3.8.1(<http://linus.nci.nih.gov/BRB-ArrayTools.html>) for subsequent microarray analyses. The data discussed in this publication have been deposited in National Center for Biotechnology Information’s (NCBI’s) Gene Expression Omnibus

(NCBIGEO GSE29703 and GSE29915). Probes with values missing from >25% of the arrays were removed from the analysis. Class comparison analysis identified microRNAs that were significantly associated with high NOS2A or CD68 mRNA expression levels. Paired class-comparison analysis identified microRNAs altered in adenomas compared to adjacent uninvolved tissue.

Reference List

1. Bartkova J, Rezaei N, Liontos M, Karakaidos P, Kletsas D et al. (2006) Oncogene-induced senescence is part of the tumorigenesis barrier imposed by DNA damage checkpoints. Nature 444: 633-637.

2. Collado M, Gil J, Efeyan A, Guerra C, Schuhmacher AJ et al. (2005) Tumour biology: senescence in premalignant tumours. Nature 436: 642.

3. Jallepalli PV, Lengauer C, Vogelstein B, Bunz F (2003) The Chk2 tumor suppressor is not required for p53 responses in human cancer cells. J Biol Chem 278: 20475-20479.

4. Bartkova J, Horejsi Z, Koed K, Kramer A, Tort F et al. (2005) DNA damage response as a candidate anti-cancer barrier in early human tumorigenesis. Nature 434: 864-870.

5. Micklem K, Rigney E, Cordell J, Simmons D, Stross P et al. (1989) A human macrophage-associated antigen (CD68) detected by six different monoclonal antibodies. Br J Haematol 73: 6-11.

6. Boersma BJ, Howe TM, Goodman JE, Yfantis HG, Lee DH et al. (2006) Association of breast cancer outcome with status of p53 and MDM2 SNP309. J Natl Cancer Inst 98: 911-919.

7. Cox GW, Mathieson BJ, Gandino L, Blasi E, Radzioch D et al. (1989) Heterogeneity of hematopoietic cells immortalized by v-myc/v-raf recombinant retrovirus infection of bone marrow or fetal liver. J Natl Cancer Inst 81: 1492-1496.

8. Dimri GP, Lee X, Basile G, Acosta M, Scott G et al. (1995) A biomarker that identifies senescent human cells in culture and in aging skin in vivo. Proc Natl Acad Sci U S A 92: 9363-9367.

9. Fujita K, Mondal AM, Horikawa I, Nguyen GH, Kumamoto K et al. (2009) p53 isoforms Delta133p53 and p53beta are endogenous regulators of replicative cellular senescence. Nat Cell Biol 11: 1135-1142.

10. Thomas DD, Espey MG, Ridnour LA, Hofseth LJ, Mancardi D et al. (2004) Hypoxic inducible factor 1alpha, extracellular signal-regulated kinase, and p53 are regulated by distinct threshold concentrations of nitric oxide. Proc Natl Acad Sci U S A 101: 8894-8899.
